# C-Terminal Tyrosine Residue Modifications Modulate the Protective Phosphorylation of Serine 129 of α-Synuclein in a Yeast Model of Parkinson's Disease

**DOI:** 10.1371/journal.pgen.1006098

**Published:** 2016-06-24

**Authors:** Alexandra Kleinknecht, Blagovesta Popova, Diana F. Lázaro, Raquel Pinho, Oliver Valerius, Tiago F. Outeiro, Gerhard H. Braus

**Affiliations:** 1 Department of Molecular Microbiology and Genetics and Göttingen Center for Molecular Biosciences (GZMB), Institute of Microbiology and Genetics, Georg-August-Universität, Göttingen, Germany; 2 Center for Nanoscale Microscopy and Molecular Physiology of the Brain (CNMPB), Göttingen, Germany; 3 Department of NeuroDegeneration and Restorative Research, University of Göttingen Medical School, Göttingen, Germany; 4 Faculty of Medicine, University of Porto, Porto, Portugal; 5 Max Planck Institute for Experimental Medicine, Göttingen, Germany; Stanford University School of Medicine, UNITED STATES

## Abstract

Parkinson´s disease (PD) is characterized by the presence of proteinaceous inclusions called Lewy bodies that are mainly composed of α-synuclein (αSyn). Elevated levels of oxidative or nitrative stresses have been implicated in αSyn related toxicity. Phosphorylation of αSyn on serine 129 (S129) modulates autophagic clearance of inclusions and is prominently found in Lewy bodies. The neighboring tyrosine residues Y125, Y133 and Y136 are phosphorylation and nitration sites. Using a yeast model of PD, we found that Y133 is required for protective S129 phosphorylation and for S129-independent proteasome clearance. αSyn can be nitrated and form stable covalent dimers originating from covalent crosslinking of two tyrosine residues. Nitrated tyrosine residues, but not di-tyrosine-crosslinked dimers, contributed to αSyn cytotoxicity and aggregation. Analysis of tyrosine residues involved in nitration and crosslinking revealed that the C-terminus, rather than the N-terminus of αSyn, is modified by nitration and di-tyrosine formation. The nitration level of wild-type αSyn was higher compared to that of A30P mutant that is non-toxic in yeast. A30P formed more dimers than wild-type αSyn, suggesting that dimer formation represents a cellular detoxification pathway in yeast. Deletion of the yeast flavohemoglobin gene *YHB1* resulted in an increase of cellular nitrative stress and cytotoxicity leading to enhanced aggregation of A30P αSyn. Yhb1 protected yeast from A30P-induced mitochondrial fragmentation and peroxynitrite-induced nitrative stress. Strikingly, overexpression of neuroglobin, the human homolog of *YHB1*, protected against αSyn inclusion formation in mammalian cells. In total, our data suggest that C-terminal Y133 plays a major role in αSyn aggregate clearance by supporting the protective S129 phosphorylation for autophagy and by promoting proteasome clearance. C-terminal tyrosine nitration increases pathogenicity and can only be partially detoxified by αSyn di-tyrosine dimers. Our findings uncover a complex interplay between S129 phosphorylation and C-terminal tyrosine modifications of αSyn that likely participates in PD pathology.

## Author Summary

Parkinson’s disease is characterized by loss of dopaminergic neurons in midbrain and the presence of αSyn protein inclusions. Human αSyn mimics the disease pathology in yeast resulting in cytotoxicity and aggregate formation. αSyn is abundantly phosphorylated at serine S129 and possesses four tyrosines (Y39, Y125, Y133, and Y136) that can be posttranslationally modified by nitration or phosphorylation. The consequence of each of these possible modifications is still unclear. Nitration as consequence of oxidative stress is a hallmark for neurodegenerative diseases. Here, we addressed the molecular mechanism, how tyrosine posttranslational modifications affect αSyn cytotoxicity. Tyrosine nitration can contribute to αSyn toxicity or can be part of a cellular salvage pathway when di-tyrosine-crosslinked dimers are formed. The Y133 residue, which can be either phosphorylated or nitrated, determines whether S129 is protectively phosphorylated and αSyn inclusions are cleared. This interplay with S129 phosphorylation demonstrates a dual role for C-terminal tyrosine residues. Yeast flavohemoglobin Yhb1 and its human counterpart neuroglobin NGB protect cells against cytotoxicity and aggregate formation. These novel insights into the molecular pathways responsible for αSyn cytotoxicity indicate NGB as a potential target for therapeutic intervention in PD.

## Introduction

Parkinson´s disease (PD) is one of the most common neurodegenerative diseases and affects about 1% of the population older than 60 years [1]. PD proceeds with selective loss of dopamine-producing neurons of the *substantia nigra pars compacta* in the ventral midbrain [2, 3]. Degeneration also occurs in other neuron types. Particularly, the mid-section of the *substantia nigra* (*zona compacta*) is affected by neurodegeneration, which is accompanied by the loss of neuromelanin pigment neurons leading to depigmentation [4, 5]. Loss of nigral dopaminergic neurons consequently results in dopamine depletion in the striatum and generates a wide range of motoric malfunctions [6]. PD is also described to be associated with non-motoric and non-dopaminergic symptoms that extend beyond the nigrostriatal dopamine pathway and often occur years or even decades prior to the clinical diagnosis [7, 8]. Typical hallmark of PD is the formation of Lewy bodies that can be observed in *post mortem* brain histology. Lewy bodies are intracellular proteinaceous inclusions with α-synuclein (αSyn) as major constituent [9–11]. Several independent point mutations in the αSyn encoding gene, as well as duplications or triplications of the wild-type αSyn locus, have been found in rare familial inherited forms of PD [12–18]. This makes αSyn a hallmark protein for PD and other related diseases, which are summarized as synucleinopathies. αSyn is a natively unfolded protein, enriched at presynaptic nerve terminals. The nuclear localization of αSyn remains under debate, since conflicting results have been obtained for the existence of αSyn in nuclei of mammalian brain neurons [19–23]. αSyn was also reported to be localized in the nucleus of cultured neurons, where it may impair histone acetylation and thereby promote neurotoxicity [24, 25]. αSyn is involved in the modulation of synaptic activity through regulation of SNARE-complex assembly of presynaptic vesicles, regulation of neurotransmitter release, regulation of cell differentiation and phospholipid metabolism [26–31].

Posttranslational modifications (PTMs) play an important role in regulating αSyn aggregation propensity and cytotoxicity. Major PTMs of αSyn include phosphorylation, ubiquitination, sumoylation or nitration [32–36]. The predominant αSyn modification is phosphorylation at serine 129 (S129). More than 90% of αSyn in Lewy bodies is phosphorylated at this residue, whereas only 4% of the soluble protein is accordingly modified [37]. The molecular function of phosphorylation at S129 is still under debate [38]. This modification modulates clearance of αSyn inclusions in a yeast model of PD [39, 40]. In addition, phosphorylation at S129 can suppress the defects induced by impaired sumoylation such as increased number of cells with inclusions and reduced yeast growth [41]. These findings support a protective function for S129 phosphorylation in this model.

Nitrated αSyn represents another PTM discovered in Lewy bodies [33, 34]. Nitration might be involved in αSyn aggregation, thereby modulating αSyn-induced cytotoxicity. Oxidative and nitrative stresses are implicated in the pathogenesis of PD [33, 34, 42–45]. Neuroinflammation followed by nitration of αSyn causes accumulation of αSyn aggregates and neurodegeneration in mice [46]. Moreover, nitrated αSyn was observed to induce adaptive immune responses that exacerbate PD pathology in the MPTP mouse model [47]. Increased nitrated αSyn is present in peripheral blood mononuclear cells of idiopathic PD patients compared to healthy individuals [48]. These studies provide evidence for a direct link between nitrative damage and the onset and progression of neurodegenerative synucleinopathies. However, the precise molecular mechanism that leads to the formation of pathological inclusions is still elusive.

Exposure of αSyn to nitrative agents results in the formation of αSyn oligomers and higher molecular weight αSyn species that are resistant to strong denaturing conditions, suggesting that αSyn proteins are covalently crosslinked [42, 49–52]. This oligomerization can be abolished *in vitro* when αSyn lacks the four tyrosine residues at positions 39, 125, 133 and 136 [53]. Three of these four tyrosine residues are located at the C-terminal end of αSyn in close neighborhood to the protective S129 phosphorylation site. Nitrating agents such as peroxynitrite/CO_2_ (PON) can nitrate tyrosine to generate 3-nitrotyrosine. Alternatively, highly stable *o*,*o’*-di-tyrosine oligomers can be formed, including dimers, trimers and higher oligomeric species [42, 54–56]. However, the majority of the studies were performed *in vitro* after exposure of αSyn to nitrating agents leading to non-specific nitration at all tyrosine residues. It is still unclear, whether the nitration-modified αSyn intermediates are toxic and what are the functional consequences of these modifications. Even the precise positions or preferred combinations for the tyrosines involved in di-tyrosine formation *in vivo* are yet unknown.

The yeast *Saccharomyces cerevisiae* is an established eukaryotic model system used to uncover the correlation between structural features of αSyn and its toxicity. It provides a unique tool to study the molecular basis of PD *in vivo* [57, 58]. 44% of the yeast genes reveal significant sequence similarities that suggest homology to human genes [59]. The basic molecular machinery necessary for neuronal function is conserved between yeast and humans. Strikingly, heterologous expression of different forms of human αSyn in yeast cells recapitulates central features of PD, including dose-dependent toxicity and aggregation. Expression of wild-type αSyn, E46K and A53T mutant results in significant growth inhibition and formation of inclusions [60, 61]. An unusual feature of the yeast system, which is different from PD and other models, is that the A30P variant only forms inclusions when highly expressed and fails to display a growth inhibition in yeast, because aggregation of A30P is only transient [62, 63]. Aggregation of αSyn in yeast cells causes mitochondrial dysfunction or formation of chemically reactive molecules, such as reactive oxygen species (ROS) and reactive nitrogen species (RNS), which is similar to mammalian cells [60, 64–69].

In this study, we analyzed the effects of nitration of wild-type and A30P mutant αSyn in yeast cells. We observed that the C-terminus of αSyn is preferentially modified by nitration compared to the N-terminus in soluble monomers as well as in dimers. Nitro- and phosphate-groups were found at Y133. Tyrosine nitration leads to increased aggregation and cytotoxicity of αSyn in yeast and confers toxicity to the non-toxic A30P mutant. The yeast nitric oxide oxidoreductase Yhb1 as well as human neuroglobin, which are encoded by homologous genes conserved from yeast to man, reduced the number of cells with inclusions and protected yeast from A30P-induced mitochondrial damage. One target of these enzymes is the C-terminal Y133 and we could show that it is required for protective phosphorylation of αSyn at S129. Our data revealed a complex choreography of posttranslational events at the αSyn C-terminus and suggest that tyrosine modifications promote subsequent S129 phosphorylation as part of a cellular control system, which contributes to the pathogenesis of the disease.

## Results

### αSyn forms dimers *in vivo*

Exposure of αSyn to nitrating agents causes tyrosine nitration *in vitro* and leads to formation of covalently crosslinked αSyn dimers and inclusions [42, 49, 50, 53, 56]. High levels of αSyn with C-terminal HIS_6_-tags were heterologously expressed in yeast cells to uncover how nitration influences *in vivo* αSyn toxicity and aggregate formation.

**Fig 1:**
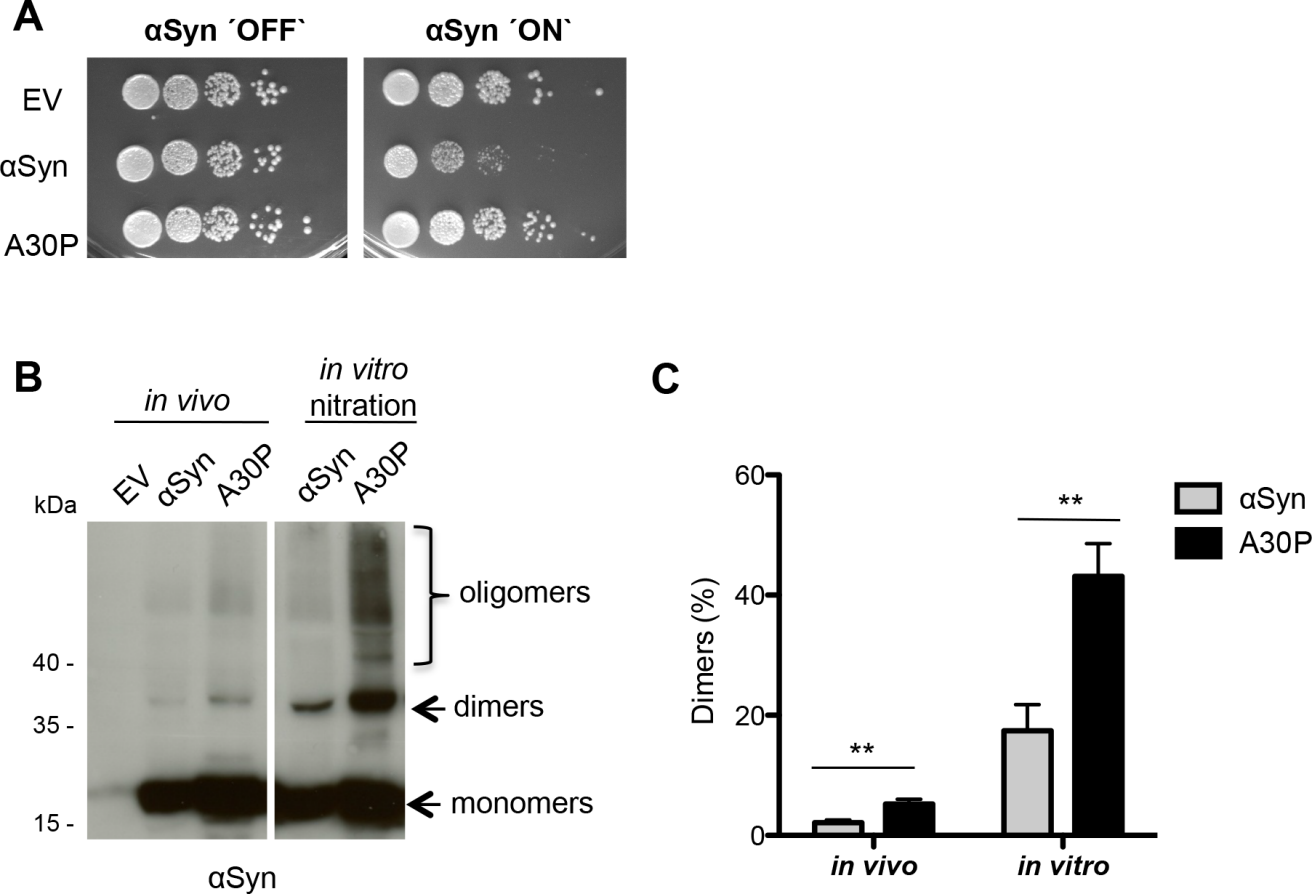
αSyn forms dimers *in vivo*. (A) Spotting analysis of yeast cells expressing C-terminally HIS_6_-tagged αSyn and A30P αSyn on a high copy vector (2μ) driven by the inducible *GAL1-*promoter on non-inducing (´OFF`: glucose) and inducing (´ON`: galactose) SC-Ura medium after 3 days. Control cells expressed only the empty vector pME2795 (EV). (B) Western blotting of αSyn and A30P enriched from cell extracts by Ni^2+^ pull-down with anti-αSyn antibody. *In vitro* nitration was carried out with 15 μg of αSyn extracts using 1 μl peroxynitrite (PON) in the presence of 1 μl 0.3 M HCl. (C) Quantification of dimers. Densitometric analysis of the immunodetection of αSyn and A30P αSyn dimers *in vivo* and in PON-treated samples. The amount of dimers is presented as percent of the total amount of αSyn detected per lane (monomer + dimer). Significance of differences was calculated with t-test (**, *p* < 0.01, n = 4).

The first approach was to examine whether αSyn and A30P form dimers *in vivo* without additional exposure of the cells to nitrating or oxidative agents. αSyn and A30P expression was driven by the *GAL1*-promoter which was repressed in the presence of glucose and induced when shifted to 2% galactose-containing medium for 12 hours (h). High copy number expression of the HIS_6_-tagged αSyn resulted in growth inhibition whereas high expression of the A30P mutant resulted in a similar growth rate as the yeast control without any αSyn (Fig 1A). Similar results were previously reported with untagged or GFP-tagged αSyn and corroborate that the HIS_6_-tag does not interfere with the behavior of αSyn in yeast [60, 63]. αSyn proteins were enriched by Ni^2+^ pull-down under denaturing conditions in the presence of urea. Immunoblotting with anti-αSyn antibody revealed distinct bands, corresponding to monomeric (~17 kDa), dimeric (~35 kDa) and higher molecular weight αSyn species (oligomers), detected from *in vivo* samples (Fig 1B). This supports that αSyn and the A30P mutant form dimers and oligomers *in vivo* even without additional exposure of the cells to nitrating or oxidative agents.

Dimer and oligomer formation of αSyn *in vivo* was further analyzed by comparison to additional *in vitro* nitration [42]. PON (ONOO^-^) was applied as nitrating agent for αSyn tyrosine residues because it leads to the formation of stable αSyn oligomers. PON is formed by the reaction of superoxide (∙O_2_^-^) with the free radical nitric oxide (∙NO). PON represents a major nitrating agent that causes tissue injury in several neurological disorders [70, 71]. αSyn and A30P proteins were expressed in yeast, pulled-down using Ni^2+^ and exposed to PON. Immunoblotting of the *in vitro* nitrated proteins revealed that the abundance of dimers and oligomers was significantly increased with the same pattern as for *in vivo* isolated αSyn species (Fig 1B). The major distinct band corresponds to the αSyn dimer species.

Quantification of the dimer band intensities of *in vivo* isolated probes revealed that A30P forms approximately twice as many dimers relative to monomers as wild-type αSyn. *In vitro* nitration of αSyn and A30P increased the total amount of dimers (Fig 1C). However, the dimer to monomer ratios between αSyn and A30P were not changed when the *in vivo* samples were enhanced by additional *in vitro* nitration (Fig 1C).

Our results suggest that the high molecular weight variants of αSyn, which can be isolated from yeast cells and which withstand strong denaturing conditions during the pull-down (8 M Urea, 2% SDS), represent covalently crosslinked αSyn species. These data support the formation of αSyn dimers in living cells. A remarkable result is that the toxicity of αSyn, which correlates to a high protein aggregation rate [63], results in a reduced amount of αSyn dimer relative to monomer formation. In contrast, the non-toxic A30P mutant that does not inhibit cellular growth (Fig 1A), and has a reduced aggregation rate, produces twice as many dimers relative to monomers in comparison to wild-type αSyn. This suggests that αSyn dimer formation is a molecular mechanism which can be used by the cell as salvage pathway for detoxification.

### The C-terminus of αSyn is preferentially modified by nitration and di-tyrosine formation

**Fig 2:**
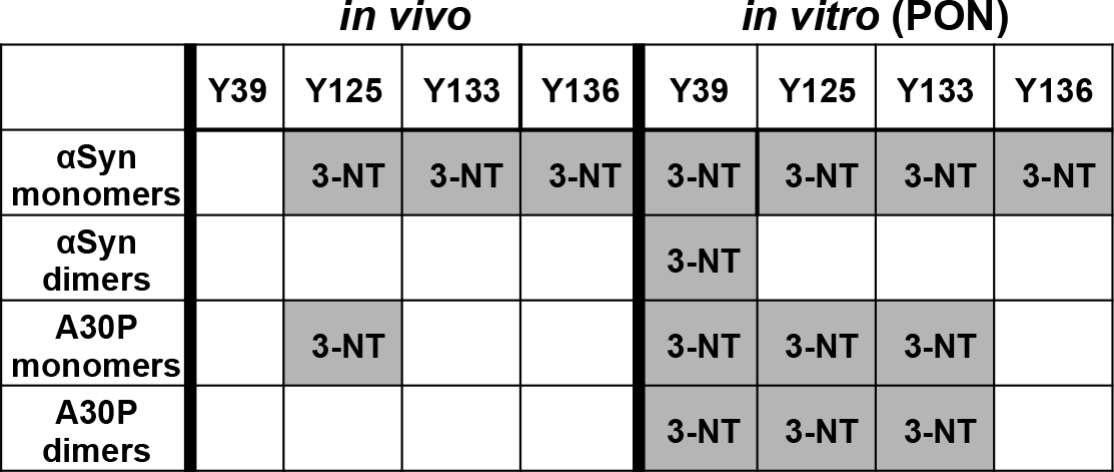
Determination of nitrated peptides from αSyn and A30P. αSyn and A30P were enriched by Ni^2+^ pull-down from yeast crude extracts and separated by SDS-PAGE. Monomeric and dimeric αSyn stained with Coomassie were excised from the gel and digested with trypsin and AspN. Untreated *(in vivo)* and subsequent peroxynitrite (PON) treated *(in vitro)* αSyn and A30P protein samples were analyzed with LC-MS for tyrosine nitration. 3-NT (3-nitrotyrosine) indicates identified nitration sites, supported by at least two peptides and two independent experiments.

Liquid chromatography–mass spectrometry (LC-MS) analysis was performed to analyze αSyn and A30P nitration sites *in vivo*. Single trypsin or AspN digestions were employed and the resulting peptides were analyzed by LC-MS. In addition to single digestions, a combined proteolytic approach by double digestion of the proteins with trypsin and AspN was employed that enabled 100% sequence coverage. The modifications of the tyrosine residues identified from fragment spectra are summarized in Fig 2. MS data revealed nitration of wild-type αSyn at all three C-terminal tyrosines (Y125, Y133, Y136). Nitration of A30P was restricted to Y125 and absent at Y133 or Y136. Nitration of the additional tyrosine residue Y39 in the N-terminal domain of αSyn could not be identified from any *in vivo* samples by MS. Pull-down and additional PON exposure, however, resulted in Y39 nitration in all samples. This suggests that Y39 is not a primary *in vivo* nitration target within cells. Additional PON-exposure after pull-down also revealed that the αSyn dimers can be potentially nitrated *in vitro*. The increased *in vitro* PON-mediated nitration of the A30P in comparison to wild-type could be due to the higher amounts of the dimer in this mutant strain.

Beyond nitration we could also identify phosphorylation in αSyn as well as A30P at S129, Y125 or Y133 but not at Y39 or Y136 (Table 1). The probabilities for possible phosphorylation sites were calculated with the phophoRS algorithm [72]. Phosphorylation of Y125 was identified with only low probability scores (Table 1). In contrast, S129 and Y133 were almost completely co-phosphorylated with scores of 100% for S129 and 99% for Y133, respectively.

**Table 1:** Phospho-peptides identified by MS/MS.

Position	Modification	PTM Score αSyn	PTM Score A30P	Sequence Motif
**Y39**	Phospho	0	1.5	KEGVL**y**VGSKT
**Y125**	Phospho	9.7	8	PDNEA**y**EMPSE
**S129**	Phospho	100	100	AYEMP**s**EEGYQ
**Y133**	Phospho	99,8	100	PSEEG**y**QDYEP
**Y136**	Phospho	0	0	EGYQD**y**EPEA

Posttranslational modification (PTM) scores were calculated with phosphoRS algorithm and represent the probability for phosphorylation modification. The corresponding amino acid is indicated by a small letter code in the sequence motif. Number of peptide sequence matches: αSyn = 332; A30P = 414.

**Fig 3:**
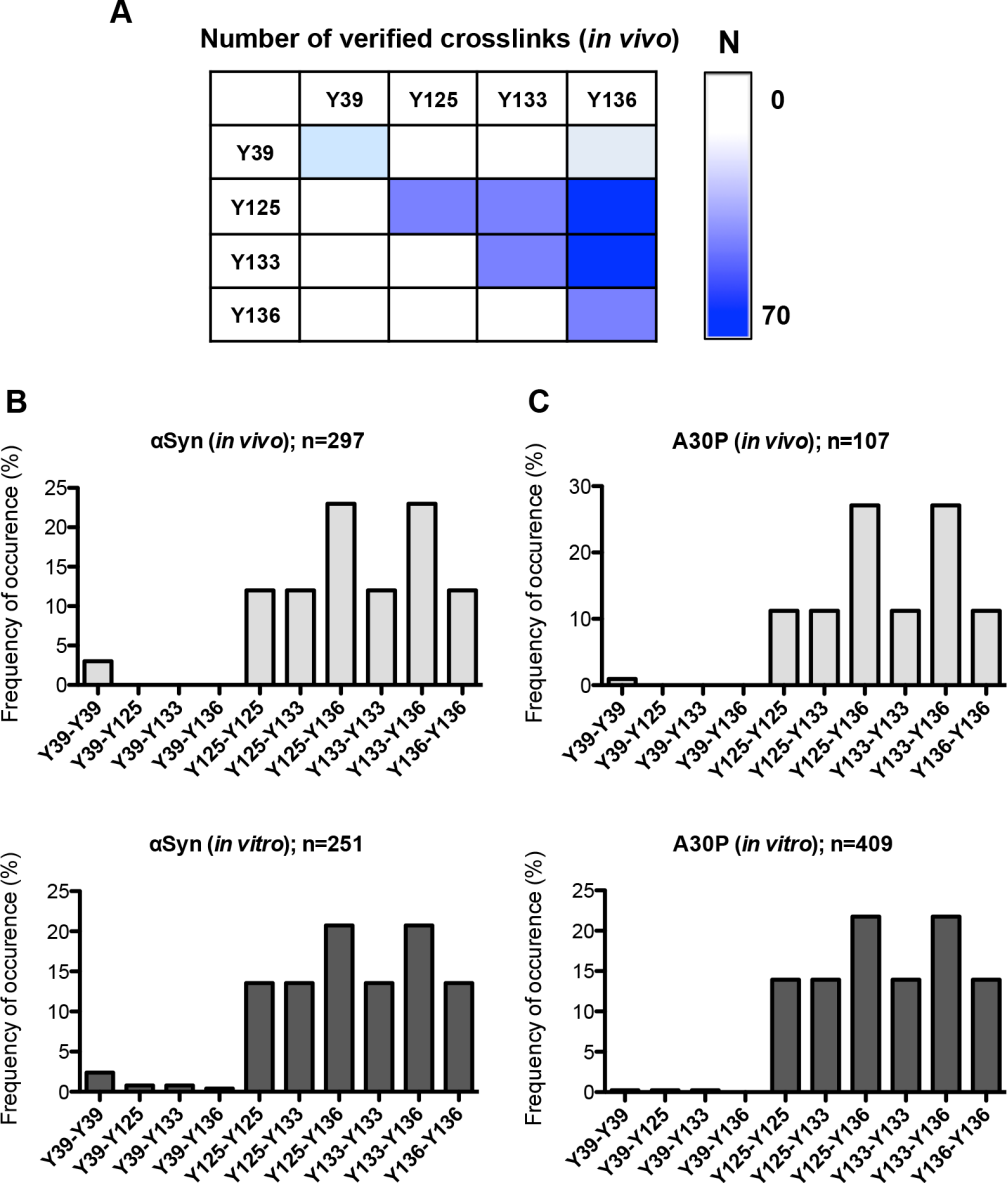
Determination of crosslinked peptides from αSyn and A30P. (A) Analysis of di-tyrosine dimers. Exemplary heat map diagram of the number (N) of identified di-tyrosine crosslinked peptides of the non-treated αSyn samples. (B) Distribution of all identified di-tyrosine peptides for αSyn. Identified combinations of crosslinked peptides are presented as percentage of n (n = total number of MS2 spectra verified as crosslinked peptides). (C) Distribution of all identified di-tyrosine peptides for A30P.

The LC-MS spectra of αSyn and A30P migrating in SDS-PAGE with the size of the dimer band were analyzed to assess whether di-tyrosines cause dimer formation of αSyn or A30P. The presence of di-tyrosine peptide crosslinks was validated using StavroX2.3.4.5 software [73]. This software compares the masses of all potential crosslinked peptides with the precursor ion masses, calculates b- and y-type ions for all possible crosslinks and compares them to MS2 data of the precursor ion. Different combinations of crosslinked peptides with an identical mass are possible when multiple tyrosine residues are located on one and the same peptide. The crosslinked tyrosine pairs were assigned according to the scores calculated by StavroX based on the fragment ion series of the MS2 spectra. The MS data analysis verified that αSyn dimers are crosslinked by tyrosine residues. The detected combinations of crosslinked tyrosines are depicted in Fig 3A. The results indicate a strong preference for crosslinking of defined combinations of tyrosines (Figs 3A, 3B, 3C and S1). The most frequent combinations for either wild-type αSyn or A30P are Y125-Y136 and Y133-Y136 dimers which are all located in the C-terminus. Only the C-terminal tyrosine residues can mutually interact. Only a small fraction of Y39-Y39 dimers were found and there are no tyrosine dimers between the N-terminal Y39 and the C-terminal tyrosines of αSyn or A30P.

These data suggest that the C-terminus of αSyn or A30P has an increased susceptibility for nitration and di-tyrosine formation compared to the N-terminus. Only Y125 is a major nitration site of A30P. In contrast, all three C-terminal tyrosines Y125, Y133 and Y136 of the wild-type αSyn are putative targets for nitration. Y133 is an additional strong and Y125 a weak phosphorylation site, respectively. Dimer formation through di-tyrosine follows a specific pattern for both tested αSyn proteins with predominant forms including Y136 interacting either with Y125 (Y125-Y136) or with Y133 (Y133-136).

### Tyrosine residues contribute to αSyn-mediated growth inhibition and aggregate formation

**Fig 4:**
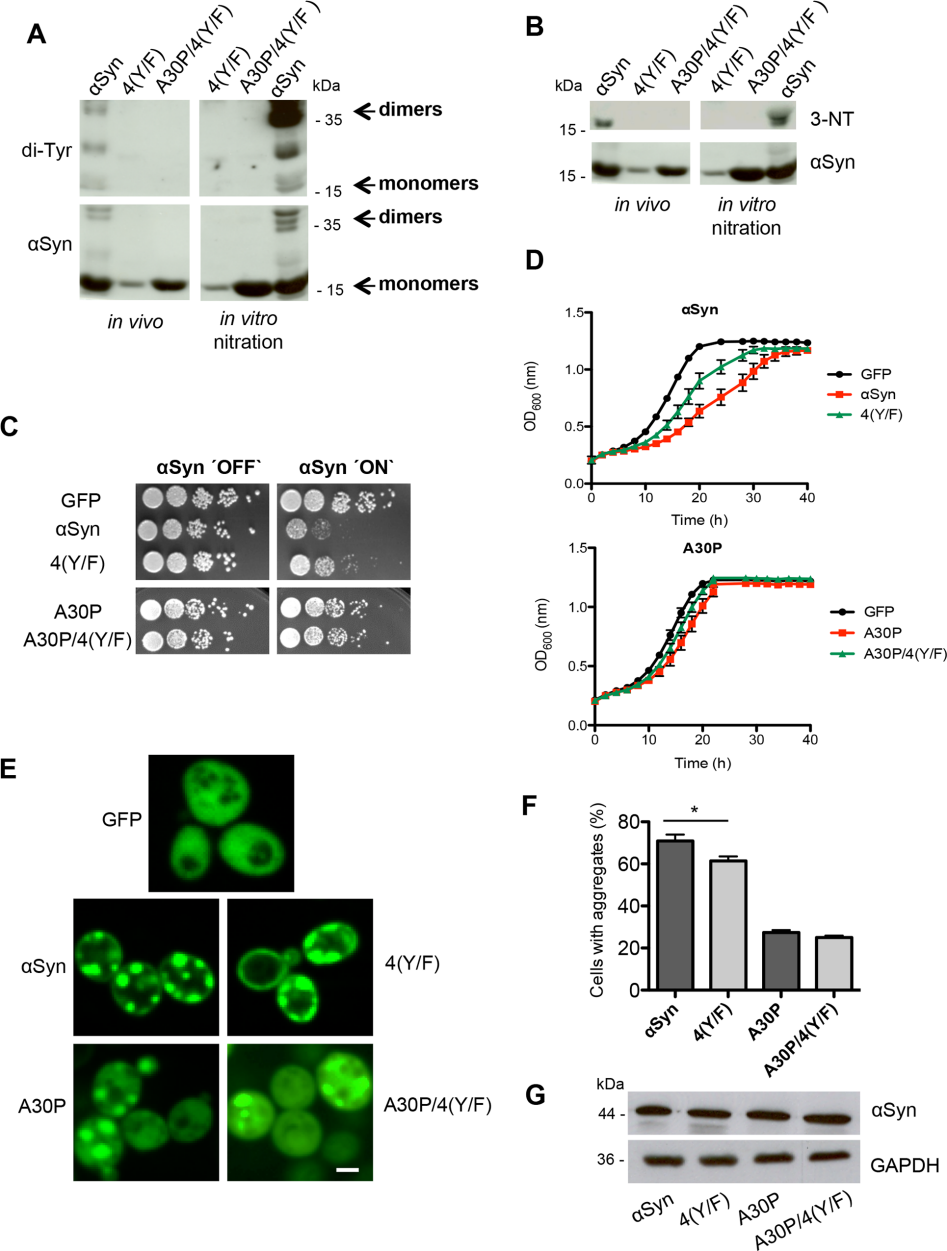
Blocking of αSyn tyrosine nitration decreases aggregation and cytotoxicity. (A) Expression of αSyn, A30P, 4(Y/F) and A30P/4(Y/F) αSyn was induced for 12 h in galactose-containing medium and the proteins were enriched by Ni^2+^ pull-down from yeast cell extracts. For *in vitro* nitration, 1 μl peroxynitrite (PON) was mixed with 15 μg of αSyn extracts in the presence of 1 μl 0.3 M HCl. Western blotting with di-tyrosine antibody reveals a major band at about 36 kDa, corresponding to dimers. Additional bands with lower molecular weights are observed, probably due to intramolecular di-tyrosine crosslinking. The same membrane was stripped and re-probed with αSyn antibody. (B) Western blotting using 3-nitro-tyrosine antibody (3-NT). Phenylalanine codon substitutions eliminate immunoreactivity. The same membrane was stripped and re-probed with αSyn antibody. (C) Spotting analysis of yeast cells expressing *GAL1*-driven αSyn, A30P, 4(Y/F), A30P/4(Y/F) αSyn and GFP (control). Yeast cells were spotted in 10-fold dilutions on SC-Ura plates containing glucose (αSyn ‘OFF’) or galactose (αSyn ‘ON’). (D) Cell growth analysis of yeast cells expressing αSyn, A30P, 4(Y/F), A30P/4(Y/F) αSyn and GFP (control) in galactose-containing SC-Ura medium for 40 h. Error bars represent standard deviations of three independent experiments. (E) Fluorescence microscopy of yeast cells, expressing indicated αSyn-GFP variants after 6 h of induction in galactose-containing medium. Scale bar: 1 μm. (F) Quantification of the percentage of cells displaying aggregates after 6 h induction in galactose-containing medium. Significance of differences was calculated with t-test (*, *p* < 0.05, n = 6). (G) Western blotting analysis of protein crude extracts of GFP-tagged αSyn, 4(Y/F), A30P and A30P/4(Y/F) after 6 h induction in galactose-containing medium. GAPDH antibody was used as loading control.

The codons for the four tyrosine sites of αSyn and A30P (Y39, Y125, Y133 and Y136) were replaced in the corresponding genes by phenylalanine codons (4(Y/F)) to analyze the role of the tyrosine residues on αSyn dimer formation, cytotoxicity or aggregation. Fusion genes with GFP-tags or HIS_6_-tags were constructed and expressed. We assessed whether the quadruple Y to F replacements influence the dimerization of αSyn and A30P. Expression of αSyn and A30P as well as their 4(Y/F) mutants was induced for 12 h. Tagged proteins were enriched by Ni^2+^ pull-down under denaturing conditions. Immunoblotting using αSyn antibodies as well as antibodies that specifically recognize di-tyrosines revealed that 4(Y/F) mutants of αSyn or A30P had lost the potential to form dimers *in vivo* (Fig 4A). Additional *in vitro* nitration with PON did also not result in any dimer or oligomer formation and served as control (Fig 4A). Immunoblotting analysis was carried out to determine *in vivo* nitrated αSyn using 3-nitro-tyrosine specific antibodies (Fig 4B). The results demonstrated that the 4(Y/F) variants of wild-type αSyn or A30P did not result in any nitration signal even after additional PON treatment. This is in contrast to wild-type αSyn with its four original tyrosine residues as control where nitration is present *in vivo* and can be further increased by additional PON treatment.

The growth impact of wild-type αSyn or the A30P variant with that of the additional 4(Y/F) substitutions were compared by spotting analysis and in liquid medium, respectively (Fig 4C and 4D). Substitutions of the four tyrosine residues in wild-type αSyn significantly improved growth on solid medium, whereas A30P αSyn growth was similar with tyrosine or instead with phenylalanine residues (Fig 4C). Growth in liquid medium resulted in similar effects, revealing significantly reduced growth inhibition of the 4(Y/F) mutant strain in comparison to wild-type αSyn, whereas A30P and its A30P/4(Y/F) derivative were growing similarly (Fig 4D).

Next, we assessed whether the decrease in wild-type αSyn toxicity was related to the formation of αSyn inclusions (Fig 4E and 4F). No change in inclusion formation could be monitored when A30P was compared to A30P/4(Y/F). However, yeast cells expressing the 4(Y/F) αSyn variant formed less inclusions in comparison to wild-type αSyn (Fig 4E and 4F). Immunoblotting with αSyn antibody revealed that the protein levels of the different αSyn variants were similar (Fig 4G).

Taken together, only tyrosine replacements by phenylalanine in case of wild-type αSyn but not in case of an additional A30P substitution reduce αSyn-induced toxicity and inclusions formation. Accordingly, there is only growth improvement in the absence of an A30P substitution that correlates with decrease of intracellular accumulations of αSyn fluorescent foci. This supports that tyrosine residues that are responsible for nitration of αSyn contribute to the cytotoxic effect and inclusion formation of αSyn in yeast. This tyrosine-dependent effect is significantly less pronounced in the presence of an A30P codon mutation suggesting that A30P suppresses the tyrosine effect, which can be observed in wild-type αSyn. The presence of tyrosine residues in wild-type αSyn favor nitration and di-tyrosine-crosslinking but offer only a minor contribution to inclusion formation.

### The nitric oxide oxidoreductase Yhb1 reduces A30P aggregation and toxicity

**Fig 5:**
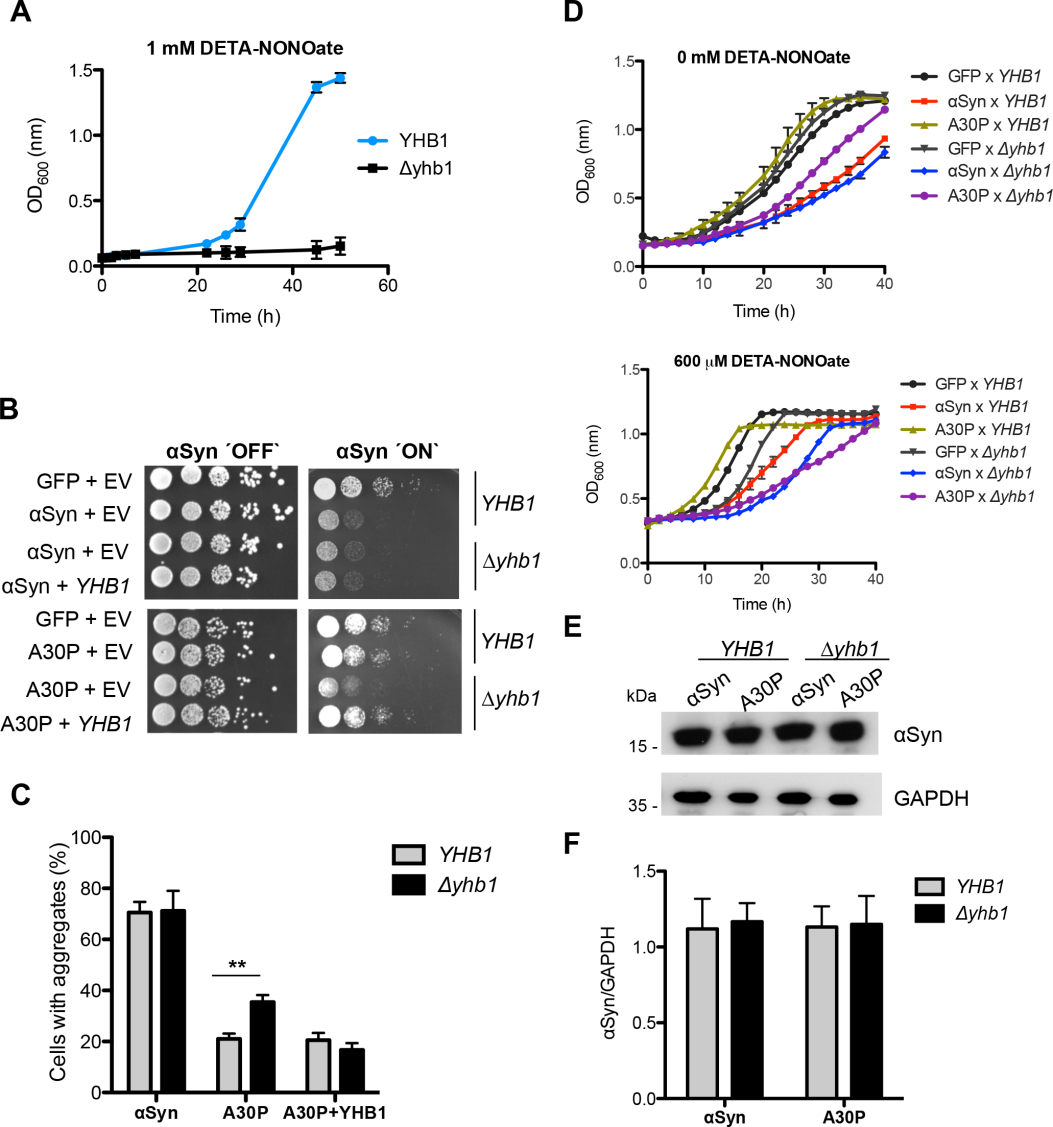
The nitric oxide oxidoreductase Yhb1 reduces A30P aggregation and toxicity. (A) Cell growth comparison of wild-type *YHB1* and mutant Δ*yhb1* yeast cells in the presence of the NO stress-mediating drug DETA-NONOate (1 mM) in liquid galactose-containing SC-Ura medium. Error bars indicate standard deviations of three independent experiments. (B) Spotting analysis of *YHB1* and Δ*yhb1* yeast cells expressing αSyn (upper boxes) or A30P (lower boxes) compared to GFP and empty vector (EV) as control on non-inducing and galactose-inducing SC-Ura medium after 3 days. (C) Quantification of the percentage of cells displaying αSyn aggregates after 6 h induction in galactose-containing medium. Significance of differences was calculated with t-test (**, *p* < 0.01, n = 6). (D) Cell growth analysis of *YHB1* and Δ*yhb1* yeast cells expressing αSyn, A30P, 4(Y/F), A30P/4(Y/F) and GFP (control) after 40 h induction in galactose-containing SC-Ura medium. (upper panel,—DETA-NONOate; lower panel, + 600 μM DETA-NONOate). Error bars show standard deviations of three independent experiments. (E) Western blotting analysis of protein crude extracts of αSyn and A30P expressed in *YHB1* and Δ*yhb1* yeast after 6 h induction in galactose-containing medium. GAPDH antibody was used as loading control. (F) Quantification of αSyn and A30P levels in *YHB1* and Δ*yhb1* yeast cells. Densitometric analysis of the immunodetection of αSyn and A30P relative to the intensity obtained for GAPDH (n = 3).

The effect of nitrative stress on the toxicity and aggregation of wild-type and A30P mutant αSyn was examined. A yeast strain carrying a deletion in the yeast flavohemoglobin gene (*YHB1*), responsible for stress signaling, was used for enhancement of nitrative stress. Yhb1 is a nitric oxide oxidoreductase, which protects against nitration of cellular targets and against cell growth inhibition under aerobic or anaerobic conditions. Deletion of *YHB1* abolishes the nitric oxide (NO) consuming activity of yeast cells [74]. The compound DETA-NONOate causes nitrative stress by acting as a NO donor. The absence of the flavohemoglobin results in a growth impairment of the hypersensitive *yhb1* deletion strain in comparison to wild-type under NO nitrative stress conditions (Fig 5A).

The genes encoding wild-type or A30P αSyn, or GFP as a control, were expressed in *Δyhb1* or the isogenic wild-type background. Cell growth was compared in the absence of nitrative stress by spotting assays (Fig 5B). Wild-type αSyn was as well cytotoxic in the presence or absence of *YHB1*. This was different for A30P, where no cytotoxicity was observed in the presence of *YHB1*. However, expression of A30P in *Δyhb1* cells inhibited cell growth. This effect was verified by low copy plasmid expression of *YHB1*. Cells rescued with *YHB1* showed the same growth phenotype as the original A30P or the GFP control in the *YHB1* background (Fig 5B).

The correlation between growth inhibition and aggregate formation of αSyn variants was examined. Cells expressing αSyn or A30P were imaged by fluorescence microscopy and the cells displaying aggregates were counted. Deletion of *YHB1* resulted in increased percentage of cells with A30P inclusions, whereas no significant difference was observed in cell expressing wild-type αSyn (Fig 5C). In agreement with the growth analysis, the complementation of the Δ*yhb1* deletion by *YHB1* rescued the lower aggregation potential of A30P.

Deletion of *YHB1* constitutes an internal stress signal. The effect of nitrative stress on A30P was further investigated by adding external nitrative stress conditions. Growth tests in liquid culture were performed using DETA-NONOate, which reduces growth of the Δ*yhb1* mutant but not of the wild-type strain (Fig 5A). Cells expressing A30P αSyn grew uninhibited in the *YHB1* wild-type background, whereas Δ*yhb1* cells expressing A30P were less inhibited than αSyn, thus recapitulating the growth phenotype in solid medium (Fig 5B and 5D). In contrast, Δ*yhb1* cells expressing both αSyn variants were equally impaired in growth under nitrative stress conditions (Fig 5D). This indicates that increase in nitrative stress changes A30P to a toxic protein in yeast cells comparable to wild-type αSyn.

αSyn toxicity is dependent on the expression levels [60, 63]. Thus, it was tested whether the A30P expression level is equal in *Δyhb1* mutant compared to *YHB1* yeast. Immunoblotting analysis revealed that the A30P variant was expressed at similar levels in both yeast backgrounds 6 h after induction of gene expression (Fig 5E and 5F), excluding that differences in toxicity are due to different A30P expression levels. These results suggest a specific suppressive function of the nitric oxide oxidoreductase Yhb1 on A30P-induced aggregate formation and growth inhibition in yeast.

### Blockade of tyrosine nitration protects against A30P toxicity and aggregate formation under nitrative stress

The removal of the four tyrosines of αSyn as possible cellular nitration sites (4(Y/F)) might affect αSyn toxicity in yeast when the intracellular nitrative stress level is increased using a Δ*yhb1* strain defective in stress protection. This was examined by comparing αSyn, A30P and their 4(Y/F) derivatives which were expressed in yeast with wild-type *YHB1* or Δ*yhb1* deletion background.

**Fig 6:**
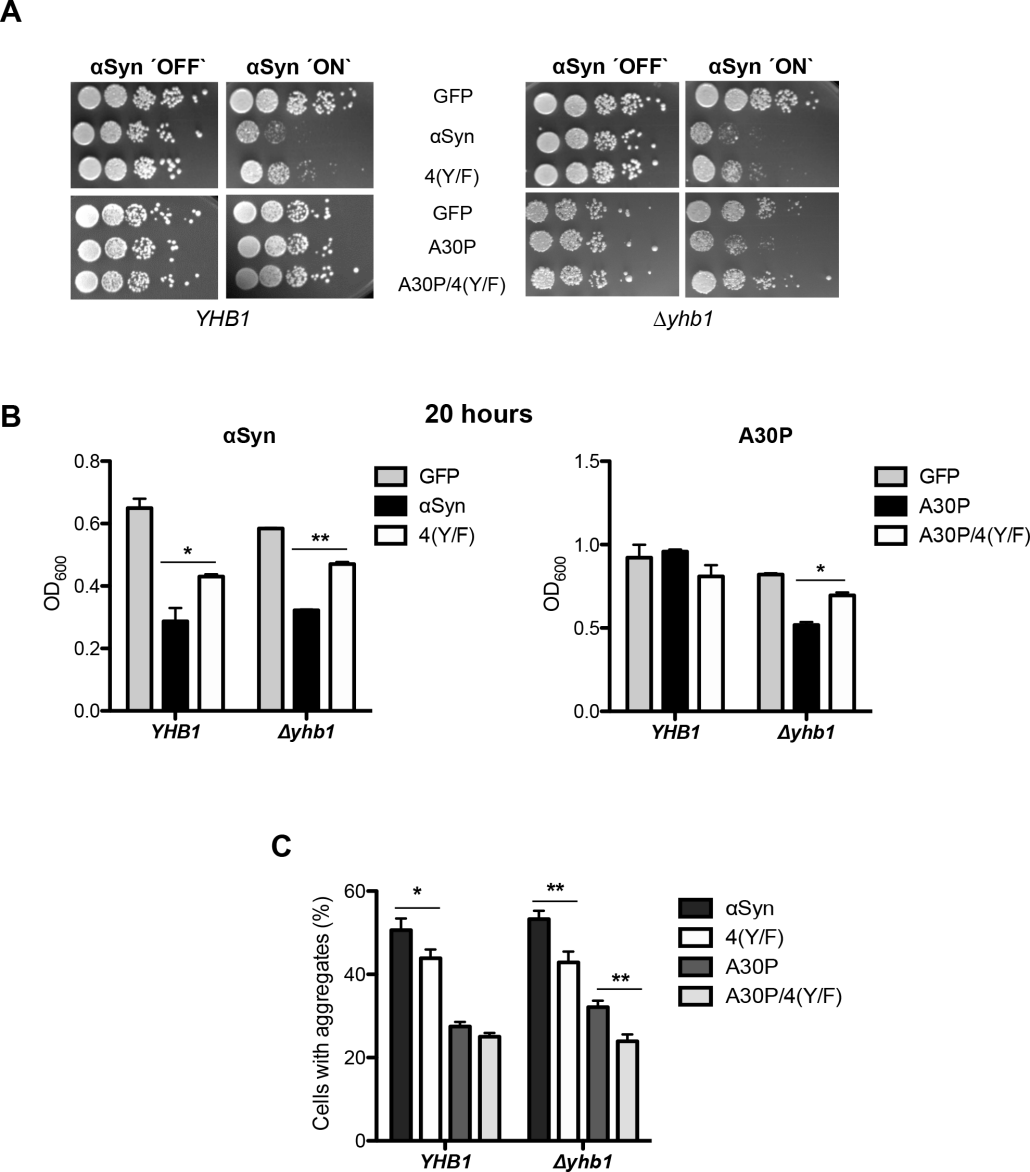
Tyrosine mutation of A30P decreases toxicity in *Δyhb1*. (A) Spotting analysis of αSyn, A30P, 4(Y/F) αSyn, A30P/4(Y/F) and GFP (control) expressed in *YHB1* and Δ*yhb1* yeast on non-inducing and galactose-inducing SC-Ura plates after 3 days of growth. (B) Cell growth analysis of *YHB1* and Δ*yhb1* yeast expressing αSyn, A30P, 4(Y/F), A30P/4(Y/F) and GFP (control) at time point 20 h. Significance of differences was calculated with t-test (*, *p* < 0.05; **, *p* < 0.01, n = 3). (C) Quantification of the percentage of cells displaying αSyn aggregates after 6 h induction in galactose-containing SC-Ura medium. Significance of differences was calculated with t-test (*, *p* < 0.05, **, *p* < 0.01, n = 6).

Growth was analyzed by spotting analysis and in liquid medium (Fig 6A and 6B). Expression of 4(Y/F) αSyn with an intact *YHB1* gene resulted in improved growth, whereas A30P toxicity was not affected (Figs 6A, 6B, 4C and 4D). In the absence of the *YHB1* gene, A30P delayed growth. However, 4(Y/F) A30P grew similar to the GFP control. A30P-mediated toxicity was related to the formation of inclusions (Fig 6C).

These results corroborate that increased nitrative stress contributes to A30P toxicity by nitration of tyrosine residues. Nitration-deficient wild-type or A30P αSyn were less toxic and aggregated less, whereas an increase of intracellular nitrative stress resulted in growth retardation and increased aggregate formation of A30P variant only when tyrosine resides were present.

### Yhb1 reduces the accumulation of reactive nitrogen species in A30P expressing yeast cells

Oxidative and nitrative stresses are implicated in the pathogenesis of PD [75, 76]. These stresses emerge from the accumulation of reactive intermediates such as ROS and RNS. ROS and RNS production were visualized in yeast cells. αSyn and A30P expression was induced for 6 h and ROS and RNS specific dyes were applied to compare the production of the reactive species in *YHB1* and Δ*yhb1* cells by fluorescence microscopy and flow cytometry.

**Fig 7:**
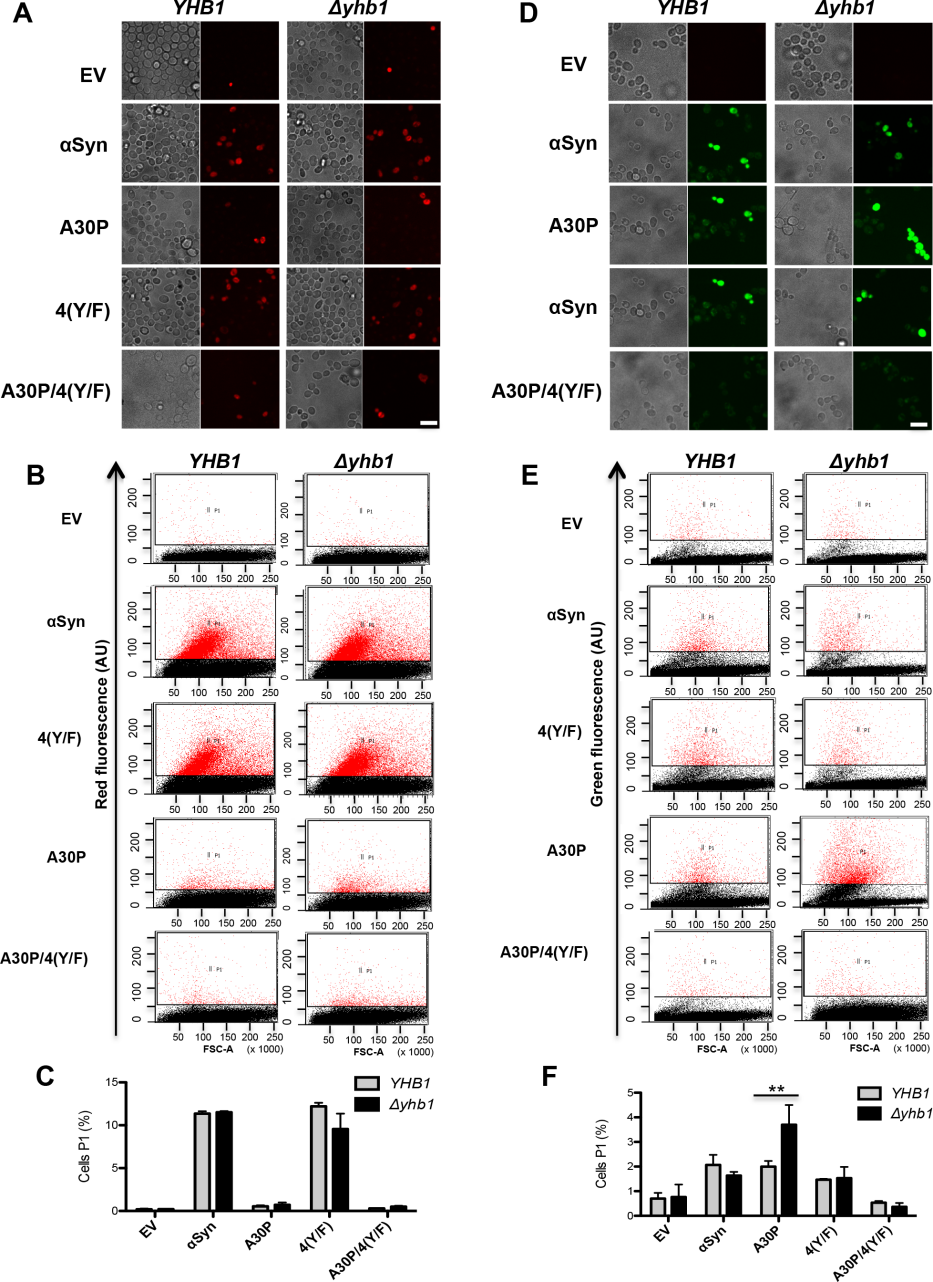
*YHB1* deletion increases accumulation of reactive nitrogen species (RNS) in A30P expressing cells. (A) αSyn, A30P, 4(Y/F) and A30P/4(Y/F) were induced in galactose-containing medium for 6 h in *YHB1* wild-type or *Δyhb1* deletion yeast strains. Cells were incubated with dihydrorhodamine 123 (DHR123) as an indicator of high intracellular ROS accumulation for 1.5 h and analyzed by live-cell fluorescence microscopy. Scale bar = 5 μm. (B) Fluorescent intensity of cells from (A), assessed with flow cytometry analysis. Forward scatter (FSC) and DHR123 fluorescence of cells after 6 h induction of αSyn expression. (C) Quantification of αSyn, A30P, 4(Y/F) and A30P/4(Y/F) expressing cells displaying ROS stained by DHR123 using flow cytometry. The percentage of the sub-population of yeast cells with higher fluorescent intensities (P1) than the background is presented. (D) Microscopy analysis of RNS stained cells. αSyn, A30P, 4(Y/F) and A30P/4(Y/F) were induced in galactose-containing SC-Ura medium for 6 h in *YHB1* and *Δyhb1* yeast strains. Cells were incubated with DAF-2 DA for 1 h at 30°C for visualization of RNS and analyzed by live-cell fluorescence microscopy. Scale bar = 5 μm. (E) Fluorescent intensity of cells from (D), assessed with flow cytometry analysis. Forward scatter (FSC) and DAF-2 DA fluorescence of cells after 6 h induction of αSyn expression. (F) Quantification of αSyn, A30P, 4(Y/F) and A30P/4(Y/F) expressing cells displaying RNS stained by DAF-2 DA using flow cytometry. The percentage of the sub-population of yeast cells with higher fluorescent intensities (P1) than the background is presented. Significance of differences was calculated with t-test (**, *p* < 0.01, n = 3).

Dihydrorhodamine 123 (DHR123) was used for ROS detection. The dye accumulates in cells, where it is oxidized by free radicals to the bright red fluorescent product rhodamine 123 (Fig 7A–7C). Expression of A30P and its derivative A30P/4(Y/F) did not significantly increase the number of cells accumulating ROS. In contrast, expression of wild-type αSyn as well as its 4(Y/F) derivative strongly increased the number of cells that accumulate red fluorescence indicative for ROS (Fig 7C). No difference in ROS accumulation was observed between the *YHB1* wild-type and the *Δyhb1* mutant strain.

DAF-2 DA (4,5-Diaminofluorescein diacetate) dye was used as a sensitive and highly specific fluorescent indicator for detection of NO (Fig 7D–7F). Expression of both αSyn and A30P induced accumulation of reactive nitrogen species (Fig 7F). Interestingly, deletion of *YHB1* significantly increased the number of cells exhibiting RNS when A30P variant was expressed. This effect was dependent on tyrosine residues since RNS accumulation in cells expressing A30P/4(Y/F) did not differ from empty vector control.

The results show that toxic wild-type αSyn expression induces significantly more ROS accumulation in yeast cells than non-toxic A30P. Accumulation of ROS species was not dependent on the presence of tyrosine residues or *YHB1* gene. In contrast, both αSyn as well as A30P induce the accumulation of RNS for nitrative stress in yeast cells. The levels of RNS in A30P but not wild-type αSyn expressing cells are dependent on the presence of tyrosine residues and *YHB1*. The results suggest that Yhb1 attenuates the accumulation of RNS of A30P expressing cells.

### Yhb1 protects mitochondria from A30P-mediated toxicity

Overexpression of αSyn and A30P leads to increased levels of RNS and higher sensitivity to NO stress in Δ*yhb1* yeast. The Yhb1 protein is translocated into yeast mitochondria under hypoxic conditions where it detoxifies NO [77]. Mitochondria are a major source of free radicals in the cells. Yhb1 is consuming NO, which inhibits mitochondrial respiration and thus increases the level of ROS. αSyn toxicity results in mitochondrial dysfunction and generation of ROS [65]. Overexpression of αSyn in mammalian cells results in mitochondrial fragmentation and involves a direct interaction of αSyn with mitochondrial membranes [78].

**Fig 8:**
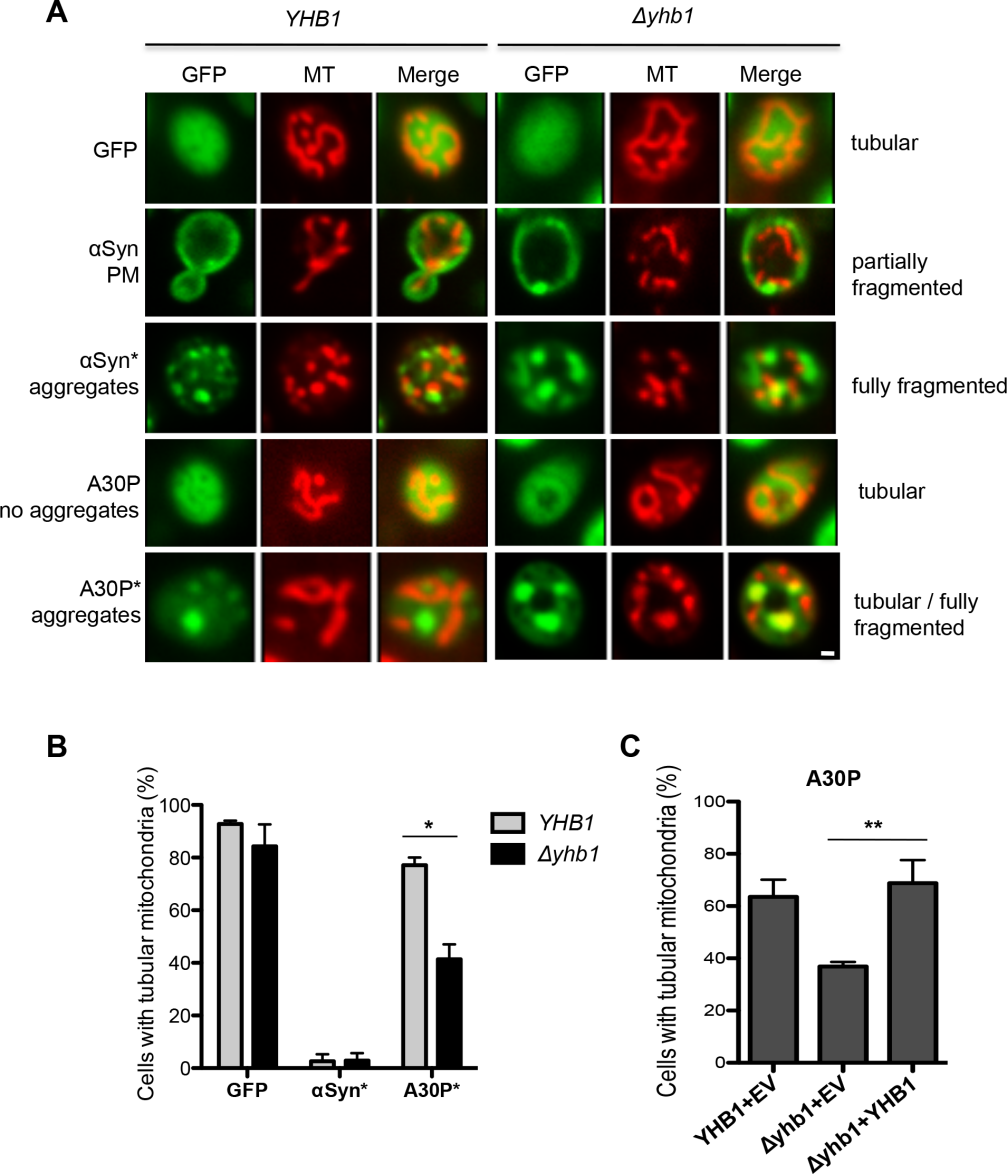
Yhb1 protects mitochondria from A30P toxicity. (A) Live-cell fluorescence microscopy of *YHB1* compared to Δ*yhb1* yeast cells expressing GFP (control), αSyn or A30P after 6 h induction in galactose-containing medium. MitoTracker Red was used to visualize mitochondria in the cells (MT panel). αSyn expressing cells with plasma membrane localization (PM) and with aggregates are visualized. Scale bar = 1 μM. (B) Quantification of yeast cells with tubular mitochondrial network. GFP: percentage of all cells with tubular mitochondria; αSyn* and A30P*: percentage of cells with aggregates, showing tubular mitochondria. At least 50 cells were counted per cell type and experiment. Significance of differences was calculated with t-test (*, *p* < 0.05, n = 4). (C) Quantification of yeast cells with tubular mitochondrial network for rescue of A30P phenotype. A30P with empty vector (EV) in *YHB1* and *Δyhb1* strain and A30P co-transformed with *YHB1* on low-copy vector in *Δyhb1* strain. A30P*: percentage of cells with aggregates, showing tubular mitochondria. Significance of differences was calculated with t-test (**, *p* < 0.01, n = 3).

We examined whether deletion of *YHB1* influences the mitochondrial morphology in αSyn and A30P αSyn expressing yeast cells. αSyn expression in the wild-type and Δ*yhb1* background was induced for 6 h in galactose medium and the mitochondria were visualized with a mitochondrial specific dye (MitoTracker Red). Cells expressing GFP were used as a control (Fig 8A). We could not detect co-localization of αSyn with mitochondria, which suggests that the described mitochondrial fraction of the protein might be small [78]. The mitochondrial morphology was classified as tubular, partially fragmented or fully fragmented. In the control cells, the mitochondria revealed a ribbon-like tubular architecture, typical for healthy mitochondria. Cells expressing αSyn showed a dramatic increase in the percentage of cells with fully fragmented mitochondria (Fig 8A and 8B). Cells with and without inclusions were considered separately for statistical evaluation. Cells with plasma-membrane localization of the GFP-signal, typical αSyn localization for early stages of expression or lower expression levels, revealed partially fragmented mitochondrial architecture. αSyn expressing cells with aggregates had fully fragmented mitochondria. In contrast to αSyn, A30P expressing cells with aggregates had mainly tubular mitochondria, similar to the control cells (Fig 8A). Deletion of *YHB1* increased the percentage of cells with fully fragmented mitochondria almost two-fold (Fig 8B). Thus, the disrupted mitochondrial morphology in Δ*yhb1* correlates with the increased levels of RNS (Fig 7D–7F) and diminished growth behavior of A30P expressing cells (Fig 5B and 5D). Complementation of the Δ*yhb1* phenotype in A30P expressing cells rescued the defect (Fig 8C), as mitochondrial morphology was recovered by expression of *YHB1* on a low-copy vector. The result suggests that Yhb1 protects against A30P-induced cytotoxicity by preventing the mitochondrial fragmentation.

### Human neuroglobin protects against αSyn aggregate formation in yeast and in mammalian cells

**Fig 9:**
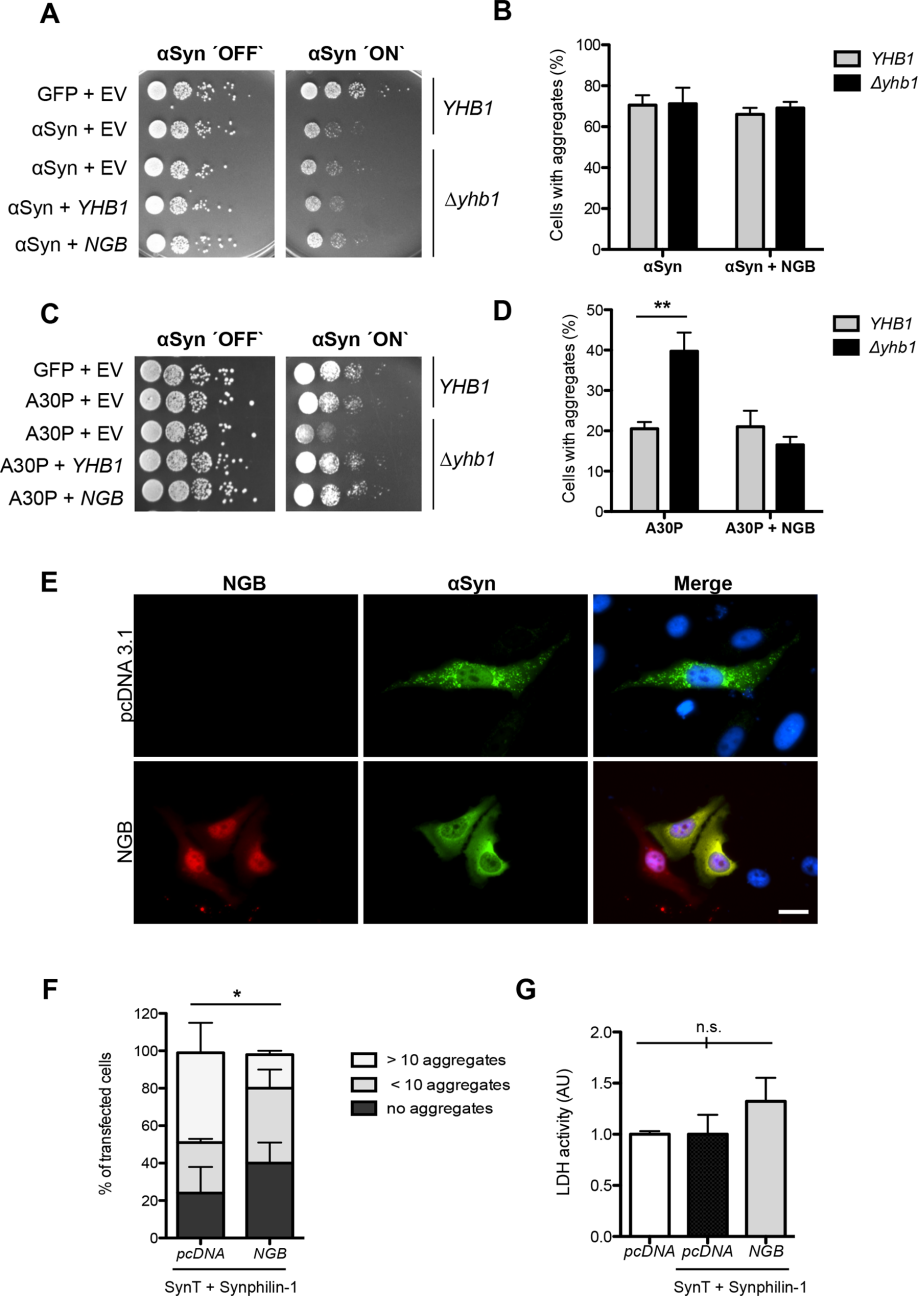
The human *NGB* gene for neuroglobin alters A30P and αSyn aggregation in yeast and mammalian cells. (A) Spotting analysis of *YHB1* and Δ*yhb1* yeast cells co-expressing αSyn and GFP (control) with either empty vector as control or *YHB1* and *NGB*, respectively, on non-inducing and galactose-inducing SC-Ura medium after 3 days. (B) Quantification of the percentage of cells displaying αSyn aggregates after 6 h induction in galactose-containing medium (n = 3). (C) Spotting analysis of *YHB1* and Δ*yhb1* yeast cells co-expressing A30P and GFP (control) with either empty vector (pME2788) as control or *YHB1* and *NGB*, respectively, on non-inducing and galactose-inducing SC-Ura medium after 3 days. (D) Quantification of the percentage of cells displaying A30P aggregates after 6 h induction in galactose-containing medium. Significance of differences was calculated with t-test (**, *p* < 0.01, n = 3). (E) Fluorescence microscopy of H4 cells co-expressing SynT, synphilin-1 and pcDNA (control) or NGB-mCherry. Nuclei are stained with Hoechst dye (blue). Scale bar = 30 μm. (F) Quantification of the percentage of H4 cells displaying αSyn inclusions after 48 h after transfection. Cells were classified into three groups according to the number of αSyn-immunoreactive inclusions observed: cells with 10 inclusions, cells with less than 10 inclusions and cells without inclusions. Significance of differences was calculated with t-test (*, *p* < 0.05, n = 3). (G) Lactate dehydrogenase (LDH) activity measurements support that *NGB* is non-toxic for H4 cells. H4 cells transfected with empty mammalian expression vector pcDNA3.1, with empty pcDNA3.1 or pcDNA3.1 encoding neuroglobin-mCherry (*NGB*) together with SynT and synphilin-1 (SynT+Synphilin-1) were analyzed. Media from indicated H4 cells were collected and the secretion of lactate LDH was determined as a measure of cytotoxicity. Significance of differences was calculated with t-test (not significant (n.s.); n = 3).

A BLAST search for human genes corresponding to yeast *YHB1* revealed 49% similarities of the *YHB1* globin domain to human neuroglobin (*NGB*) as a putative homolog. We analyzed whether the human counterpart of yeast *YHB1* can affect αSyn aggregation. Neuroglobins are oxygen-binding proteins that are highly conserved among vertebrates and are expressed in the central and peripheral nervous system. They provide protection against hypoxic induced cell injury in the brain, which is associated with ROS and RNS accumulation [79]. Both Yhb1 and neuroglobin contain a globin domain and are members of the globin gene family. *NGB* was shown to diminish beta-amyloid-induced neurotoxicity *in vitro* and to attenuate the phenotypes in a transgenic mouse model of Alzheimer’s disease [80] and to act as an oxidative stress-responsive sensor for neuroprotection [81]. Here we examined, whether human *NGB* affects αSyn or A30P growth and aggregate formation in yeast. Growth and aggregation of αSyn was not changed by the expression of the human *NGB* (Fig 9A and 9B). However, *NGB* expression in Δ*yhb1* deletion strain rescued A30P yeast growth (Fig 9C) and reduced the number of cells with A30P aggregates (Fig 9D). The effect of *NGB* in yeast is similar to the impact of *YHB1* on αSyn and A30P growth and aggregate formation (Fig 5C).

Next, we examined whether *NGB* has not only a protective role against αSyn aggregate formation in yeast but also in mammalian cells. Human Neuroglioma cells (H4) served as established αSyn aggregation model, where aggregation of αSyn is induced by co-expressing C-terminally modified αSyn (SynT) and synphilin-1, αSyn-interacting protein that was also found in LBs [61, 82]. H4 cells were co-transfected with SynT, synphilin-1 and *NGB* or empty vector and aggregate formation of SynT was monitored (Fig 9E and 9F). Expression of *NGB* reduced the number of cells with aggregates almost two-fold in comparison to the control and reduced the number of aggregates per cell. Lactate dehydrogenase (LDH) measurements were performed to determine, whether there is an effect of *NGB* on cell toxicity. LDH release into the cell culture medium is an indicator of damages of the plasma membrane and is used as cytotoxicity marker. LDH measurements were similar for all tested H4 cells and support that *NGB* does act as suppressor of αSyn aggregation without significantly causing cytotoxicity.

### Yhb1 affects nitration but not dimerization level of αSyn and A30P

**Fig 10:**
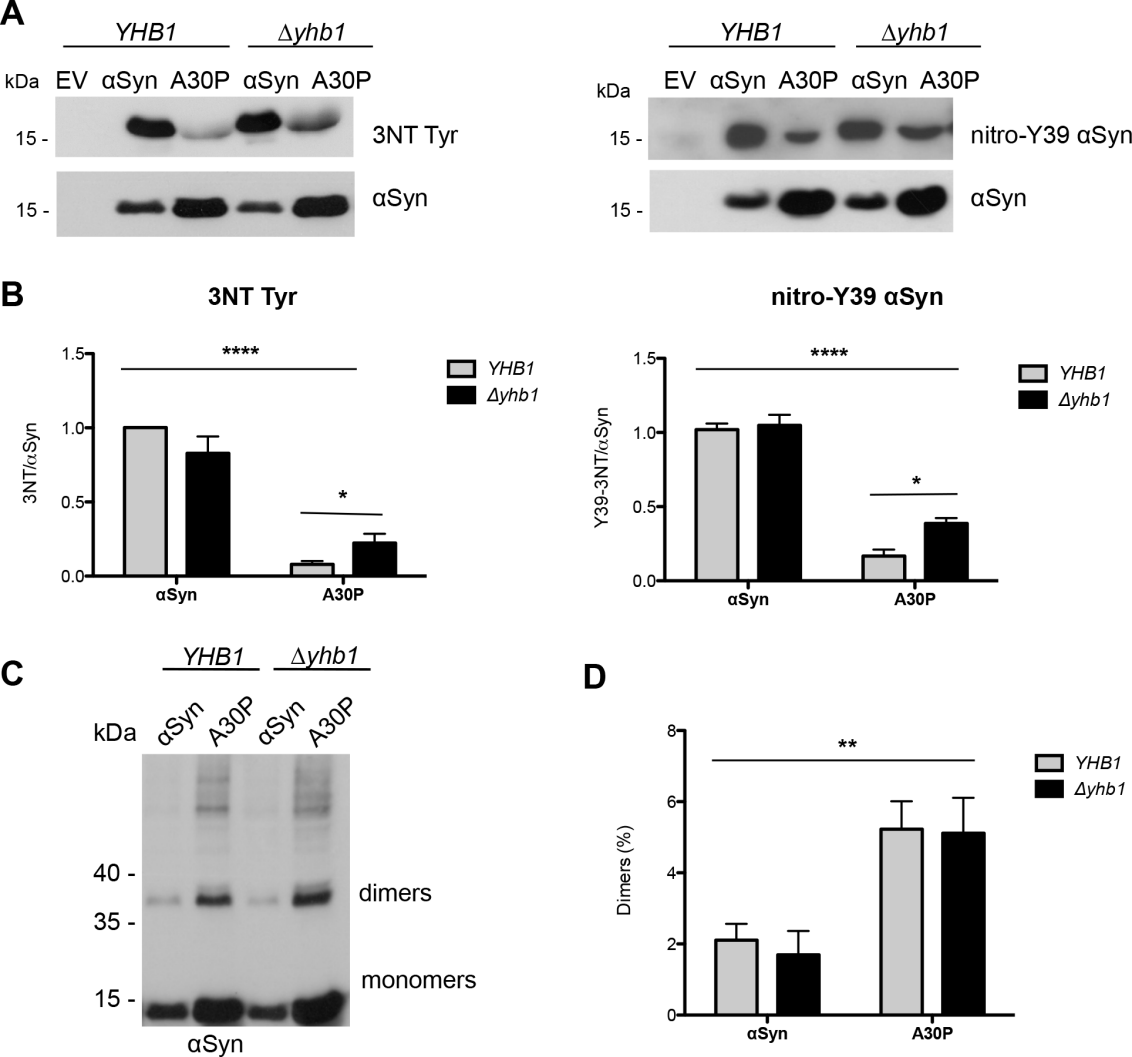
Yhb1 affects nitration but not dimerization of αSyn and A30P. (A) Immunoblotting analysis of 3-nitrotyrosine using 3-nitrotyrosine antibody (left) and nitro-Y39 αSyn antibody (right). Protein expression was induced for 12 h in galactose-containing SC-Ura medium. Concentrated protein extracts of Ni^2+^ pull down-enriched αSyn and A30P αSyn from *YHB1* and *Δyhb1* yeast cells were applied. Cells expressing empty vector (EV) served as control. The same membranes were stripped and re-probed with αSyn antibody. (B) Quantification of αSyn and A30P nitration levels in *YHB1* and Δ*yhb1* yeast cells. Densitometric analysis of the immunodetection of nitrated αSyn and A30P relative to the intensity obtained for αSyn. Significance of differences was calculated with one-way ANOVA with Bonferroni’s multiple comparison test (*, *p* < 0.05; ****, *p* < 0.0001; n = 3). (C) Western blotting of αSyn enriched by Ni^2+^ pull-down with αSyn antibody. (D) Ratio of dimers relative to the sum of monomers and dimers. Densitometric analysis of the immunodetection of αSyn and A30P αSyn dimers, presented as percent of the total amount of αSyn detected per lane (monomer + dimer). Significance of differences was calculated with one-way ANOVA (**, *p* < 0.01; n = 4).

We assessed whether the different cytotoxicity of αSyn and A30P in yeast correlates with different nitration levels of the two variants in wild-type yeast background and under increased nitrative stress in Δ*yhb1* strain. Immunoblotting analysis performed with two specific antibodies against nitro-tyrosine (3-nitrotyrosine and nitro-Y39 αSyn) showed that αSyn and A30P are nitrated in the *YHB1* as well as in *Δyhb1* yeast (Fig 10A). Quantification of band intensities of both nitrated αSyn variants revealed significantly higher nitration level of αSyn in comparison to A30P. Deletion of *YHB1* resulted in increase of A30P nitration level, whereas αSyn nitration level was not affected (Fig 10B). The increased nitration level does not correlate with an increased dimerization level of both αSyn variants. The dimerization was not influenced by nitrative stress enhancement (Fig 10C and 10D). The results suggest that nitration of αSyn contributes to the cytotoxicity of the protein, whereas dimer formation is in reverse correlation to toxicity.

### Tyrosine 133 is required for phosphorylation of αSyn at serine 129

Phosphorylation of Y125 is required *in vitro* as a priming event for efficient phosphorylation of S129 by casein kinase CK1 [83]. Phosphorylation of S129 is the major PTM of αSyn, found in 90% of the aggregated protein in neuronal inclusions of PD patients [37]. αSyn and A30P are phosphorylated at S129 in yeast by endogenous kinases and phosphorylation has a protective role against αSyn-induced toxicity and aggregate formation [39, 41]. Given the importance of these PTM and the close proximity of serine and tyrosine residues at the C-terminus, we assessed whether there is a cross-talk between modifications of tyrosine residues and phosphorylation of αSyn at S129 *in vivo*.

**Fig 11:**
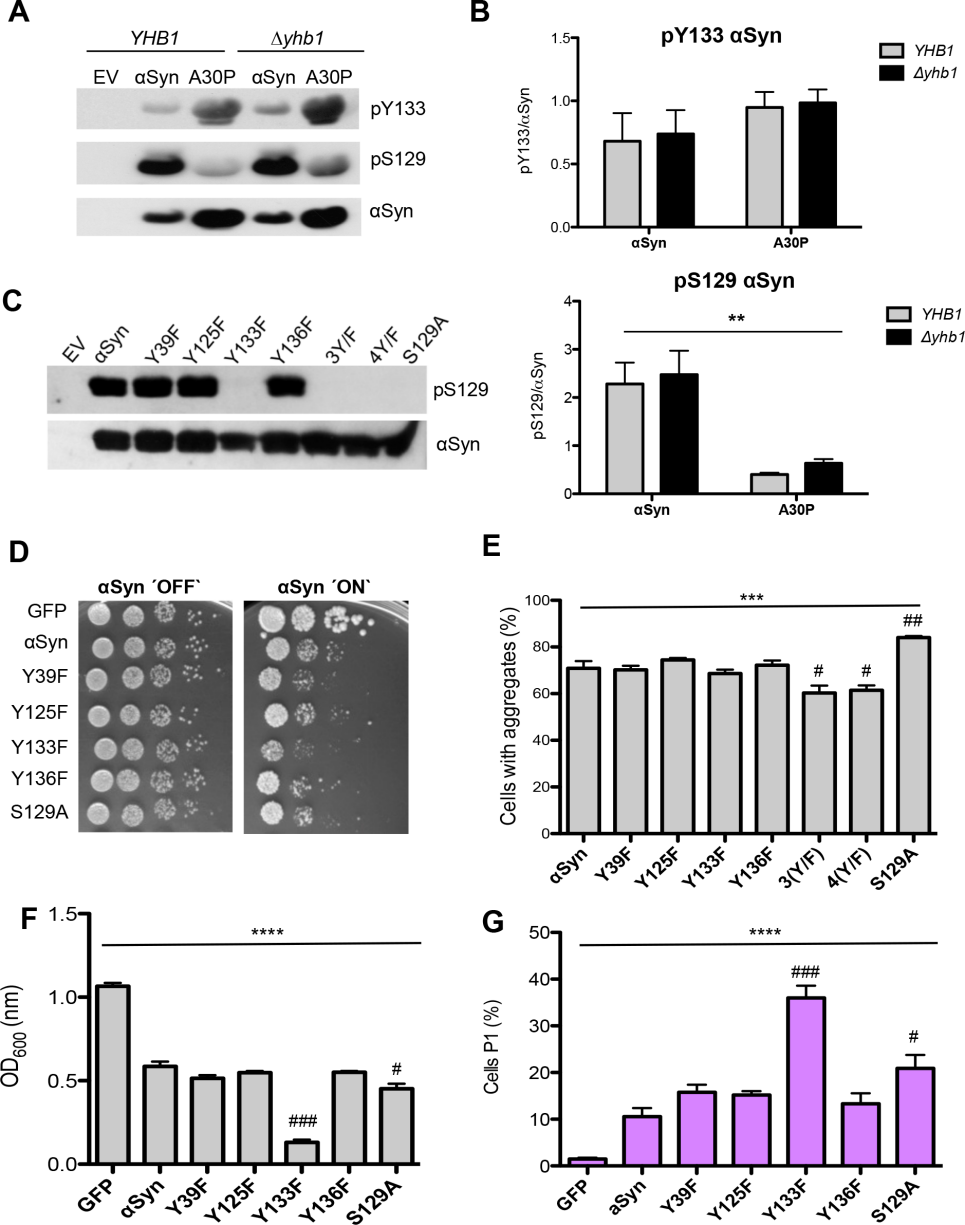
Tyrosine 133 is required for phosphorylation of αSyn at serine 129. (A) Western blotting of αSyn and A30P expressed in *YHB1* and Δ*yhb1* yeast enriched by Ni^2+^ pull-down, using Y133 phosphorylation-specific αSyn antibody (pY133) and S129 phosphorylation-specific αSyn antibody (pS129). The same membrane was stripped and re-probed with αSyn antibody. (B) Quantification of αSyn and A30P Y133- and S129-phosphorylation levels in *YHB1* and Δ*yhb1* yeast cells. Densitometric analysis of the immunodetection of pY133, pS129 αSyn and A30P relative to the intensity obtained for αSyn. Significance of differences was calculated with one-way ANOVA test (**, *p* < 0.01; n = 4). (C) Western blotting of crude extracts from yeast cells, expressing different αSyn variants after 6 h induction of protein expression using S129 phosphorylation-specific αSyn antibody (pS129) and αSyn antibody. Cells expressing S129A mutant served as control. (D) Spotting analysis of αSyn and indicated mutant strains, driven by the inducible *GAL1-*promoter on non-inducing (´OFF`: glucose) and inducing (´ON`: galactose) SC-Ura medium after 3 days. Cells expressing GFP served as control. (E) Quantification of the percentage of cells displaying αSyn aggregates after 6 h induction in galactose-containing SC-Ura medium. Significance of differences was calculated with one-way ANOVA (***, *p* < 0.001) or Dunnett’s multiple comparison test (#, *p* < 0.05, ##, *p* < 0.01 versus αSyn; n = 6). (F) Cell growth analysis of cells expressing different αSyn variants and GFP (control) after 20 h induction of expression. Significance of differences was calculated with one-way ANOVA (****, *p* < 0.0001) or Dunnett’s multiple comparison test (#, *p* < 0.05; ###, *p* < 0.001, n = 4). (G) Quantification of cells expressing different αSyn variants and GFP (control) displaying Propidium Iodide (PI) fluorescence after 20 h induction of αSyn expression, assessed by flow cytometry. The percentage of PI-positive yeast cells with higher fluorescent intensities (P1) than the background is presented. Significance of differences was calculated with one-way ANOVA (****, *p* < 0.0001) or Dunnett’s multiple comparison test (#, *p* < 0.05; ###, *p* < 0.001 versus αSyn; n = 4).

Immunoblotting with an antibody that specifically recognizes αSyn phosphorylated at Y133 showed that both αSyn and A30P are phosphorylated at these residues, in accordance with our results from MS analysis (Fig 11A). Quantification of Y133 phosphorylation revealed similar phosphorylation level of αSyn and A30P variant both in presence and absence of Yhb1 (Fig 11B). We analyzed whether there is a difference between S129 phosphorylation level of αSyn and A30P. S129 phosphorylation level of αSyn was much higher than that of A30P (Fig 11A and 11B). Tyrosine to phenylalanine (Y/F) substitutions were analyzed for their effects on the phosphorylation level at S129. Y/F mutation of the N-terminal tyrosine 39 as well as of the C-terminal Y125 and Y136 did not affect the phosphorylation status of S129. In contrast, mutation of Y133 had a drastic impact and resulted in complete loss of phosphorylation at S129 (Fig 11C). Yeast growth was compared in spotting assay as well as in liquid culture between yeast cells, expressing Y/F single mutants and S129A phosphorylation deficient mutant (Fig 11D and 11F). Yeast growth was measured after 20 h induction of protein expression. Expression of Y133F resulted in significant growth inhibition in comparison with αSyn and other tyrosine mutants. S129A showed slight growth inhibition (Fig 11D and 11F) and significant increase in the number of cells with aggregates (Fig 11E). In addition to growth analysis, membrane integrity of the Y/F single mutants and S129A phosphorylation deficient mutant was examined to assess the cell viability of the mutants (Fig 11G and S2 Fig). Propidium iodide (PI) staining was used after 20 h of protein induction as a sensitive method to determine the fraction of cells with compromised membrane integrity. Expression of Y133F and S129A significantly diminished membrane integrity, corroborating that expression of these mutants results in increased cytotoxicity.

**Fig 12:**
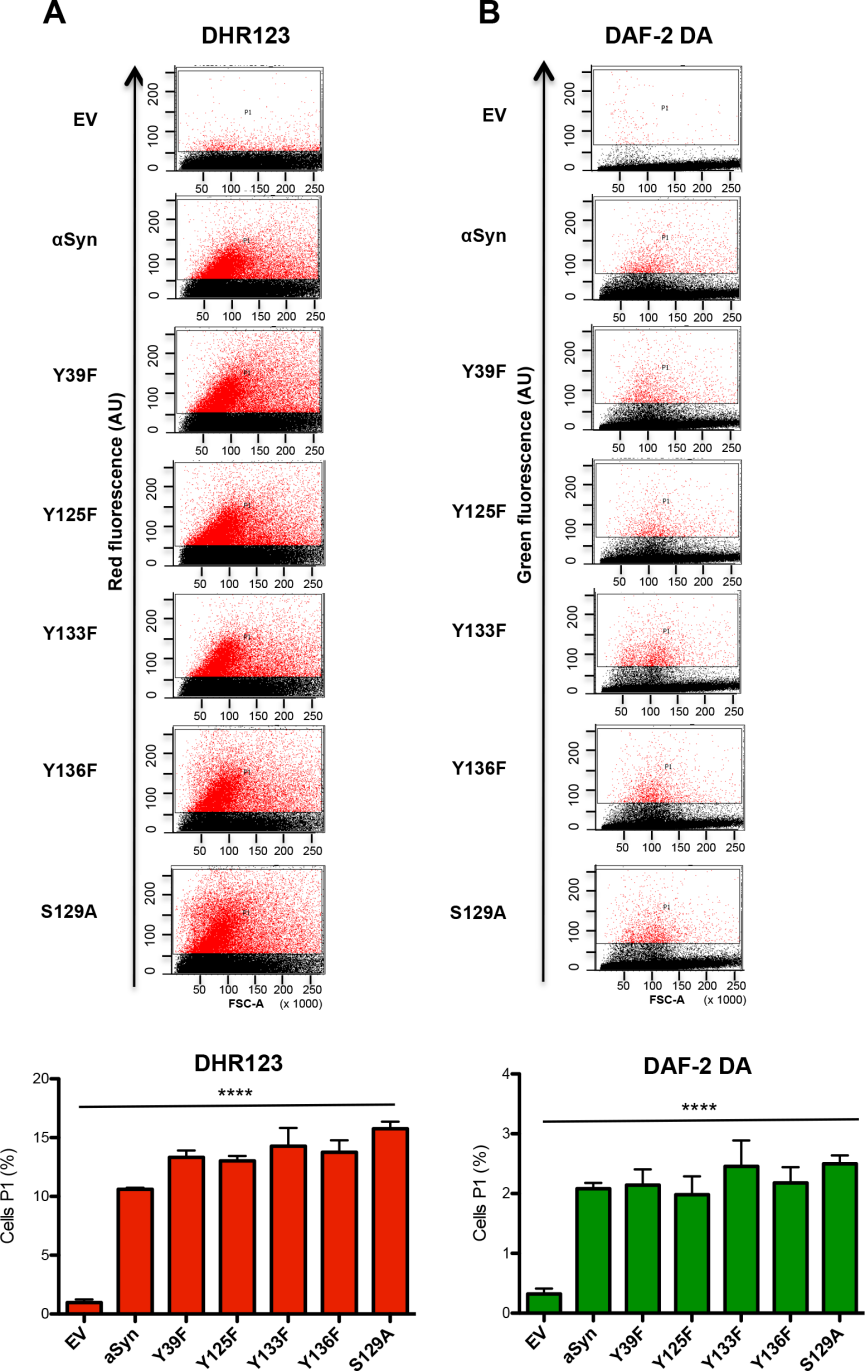
Tyrosine 133 mutation does not alter the accumulation of reactive oxygen and nitrogen species. (A, B) Quantification of cells expressing different αSyn variants displaying ROS and RNS assessed with flow cytometry analysis. αSyn expression was induced for 6 h and the cells were stained for 1.5 h with DHR123 to visualize ROS (A) or with DAF-2 DA to visualize RNS (B). Forward scatter (FSC) and DHR123 (A) or DAF-2 DA (B) fluorescence of the cells, showing one representative result from at least four independent experiments. The percentage of the sub-populations of yeast cells with higher fluorescent intensities (P1) than the background are presented in the lower panels. Significance of differences was calculated with one-way ANOVA (****, *p* < 0.0001).

Flow cytometry measurements were performed to determine the accumulation of ROS and RNS in yeast cells, expressing the single mutants. DHR123 was used for detection of ROS (Fig 12A) and DAF-2 DA was used for detection of RNS (Fig 12B). Expression of all mutants revealed a significant increase in the levels of ROS and RNS in comparison with the control; however, no significant differences were observed between the single mutants revealing that the enhanced toxicity of Y133F and S129A mutant is not due to higher accumulation of ROS or RNS.

These results indicate that Y133 is required for the protective effect of αSyn S129 phosphorylation *in vivo*. Expression of Y133F was more toxic than expression of S129A phosphorylation deficient mutant, suggesting additional protective contribution of Y133 modifications against αSyn cytotoxicity. The data support a complex cross-talk between nitration and phosphorylation of the C-terminal tyrosine residues and S129 phosphorylation of αSyn and A30P.

### C-terminal αSyn modifications promote autophagy clearance of αSyn aggregates

**Fig 13:**
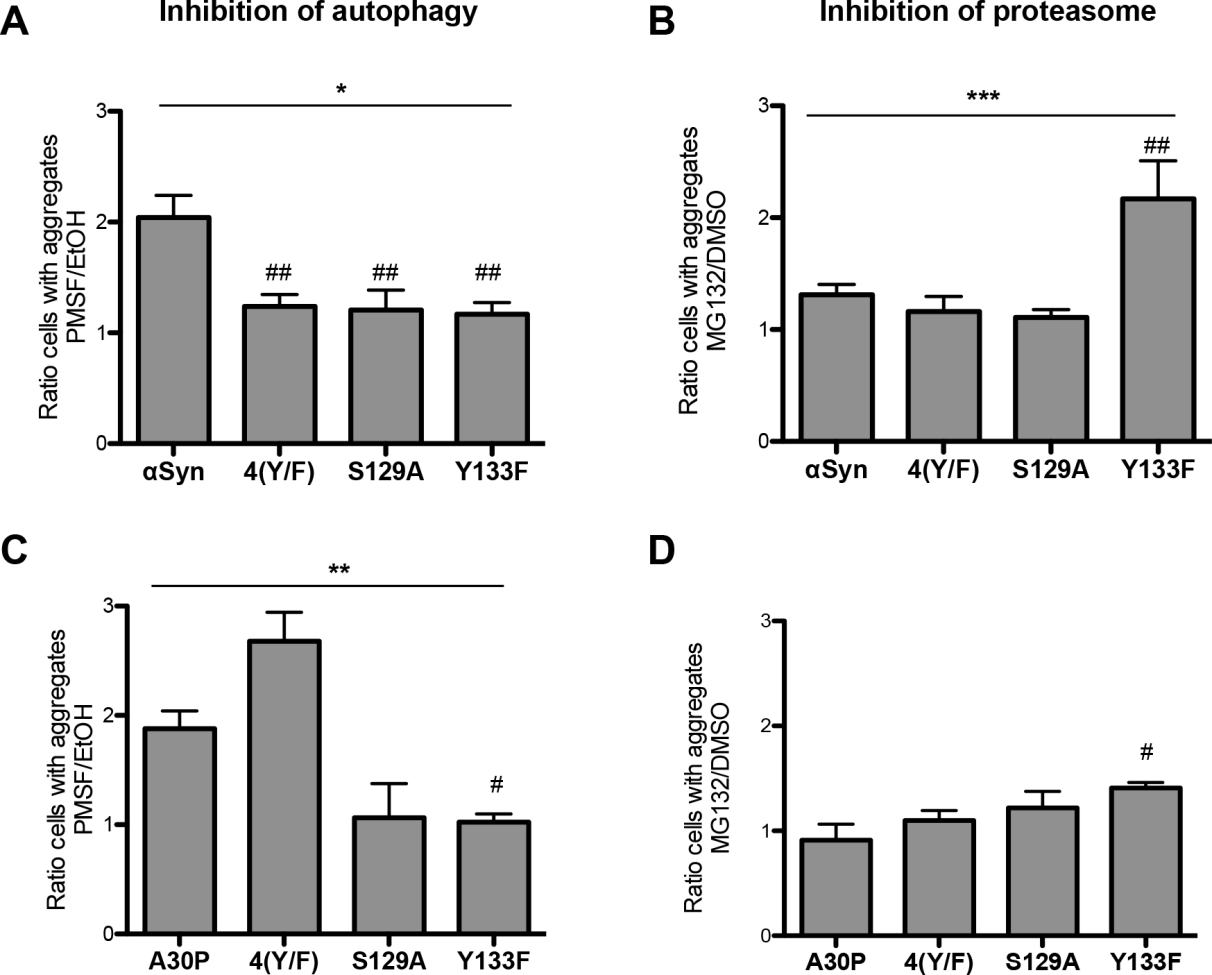
αSyn aggregate clearance after promoter shut-off. (A, C) Quantification of cells displaying aggregates of αSyn (A) and A30P (C) upon inhibition of autophagy by PMSF. Cells expressing αSyn (A) or A30P (C) and its 4(Y/F), S129A and Y133F variants were incubated in 2% galactose-containing media for 4 h and shifted to 2% glucose-containing media supplemented with 1 mM PMSF dissolved in EtOH and only EtOH as a control. Cells with aggregates were counted after 4 h *GAL1*-promoter shut-off and presented as a ratio to the control (EtOH). Significance of differences was calculated with one-way ANOVA (*, *p* < 0.05; **, *p* < 0.01) or Dunnett’s multiple comparison test (#, *p* < 0.05; ##, *p* < 0.01 versus αSyn; n = 4). (B, D) Quantification of cells displaying aggregates of αSyn (B) and A30P (D) upon inhibition of the proteasome by MG132. Cells expressing αSyn (B) or A30P (D) and the indicated 4(Y/F), S129A and Y133F variants were incubated in 2% galactose-containing media for 4 h and shifted to glucose medium, supplemented with 75 μM MG132, dissolved in DMSO or only DMSO as a control. Cells with aggregates were counted after 4 h *GAL1*-promoter shut off and presented as a ratio to the control (DMSO). Significance of differences was calculated with one-way ANOVA (***, *p* < 0.001) or Dunnett’s multiple comparison test (#, *p* < 0.05; ##, *p* < 0.01 versus αSyn; n = 4).

*GAL1* promoter shut-off experiments were performed to study the role of αSyn PTMs on autophagy/vacuole and proteasome-mediated aggregate clearance of αSyn. The impact of blocking these systems by drug treatments was examined. Expression of αSyn was induced for 4 h in galactose-containing medium and the cells were then shifted to glucose-containing medium in order to repress the promoter. Cells were imaged 4 h after promoter shut-off and the percentage of cells with inclusions was determined. Shut-off studies were performed with wild-type αSyn and the mutants 4(Y/F), S129A and Y133F. PMSF was used as an inhibitor of autophagy/vacuole to study the contribution of this pathway for aggregate clearance [63]. PMSF impairs the activity of many vacuolar serine proteases without affecting proteasome function [84, 85]. Inhibition of autophagy resulted in inefficient aggregate clearance of αSyn, as shown previously [41, 63]. Mutations of the codons for the four tyrosines as well as the S129 and Y133 single exchanges resulted in similar aggregate clearance by inhibition of the autophagic proteases as in the control cells without drug (ethanol) (Fig 13A). This suggests that autophagy is less involved in aggregate clearance of these mutants and shows that autophagy-mediated aggregate clearance requires modifications of the tyrosines and S129. The contribution of the proteasome on 4(Y/F), S129A and Y133F αSyn aggregate clearance was analyzed by applying the proteasome inhibitor MG132 [86]. In contrast to autophagy impairment, cells expressing 4(Y/F) and S129A αSyn cleared inclusions equally as the wild-type αSyn (Fig 13B) when the proteasome system was impaired. These results corroborate our previous findings showing a minor contribution of the proteasome to αSyn aggregate clearance [63]. However, cells expressing the Y133F mutant were unable to clear inclusions in a same manner as the wild-type, 4(Y/F) and S129A αSyn. This indicates that αSyn, which is not modified at Y133, is degraded by the proteasomal pathway. The results suggest that PTMs of tyrosine residues and S129 promote the autophagy-mediated aggregate clearance, whereas non-modified Y133 residue is a key determinant for the targeting of the protein to the proteasome.

Inhibition of autophagy of A30P expressing cells revealed diminished clearance of aggregates of A30P as well as the A30P/4(Y/F) mutant indicating that degradation of the A30P/4(Y/F) aggregates depends on the autophagy/vacuole system similarly to wild-type αSyn and A30P (Fig 13C). A30P/S129A and A30P/Y133F mutants were able to degrade aggregates efficiently upon autophagy inhibition, similar to S129A and Y133F. Proteasome impairment resulted in inefficient clearance of the A30P/Y133F mutant (Fig 13D). However, this impact was not as strong as in the αSyn Y133F mutant, confirming that the wild-type αSyn is strongly dependent on Y133 modification as a determinant for aggregate clearance.

## Discussion

**Fig 14:**
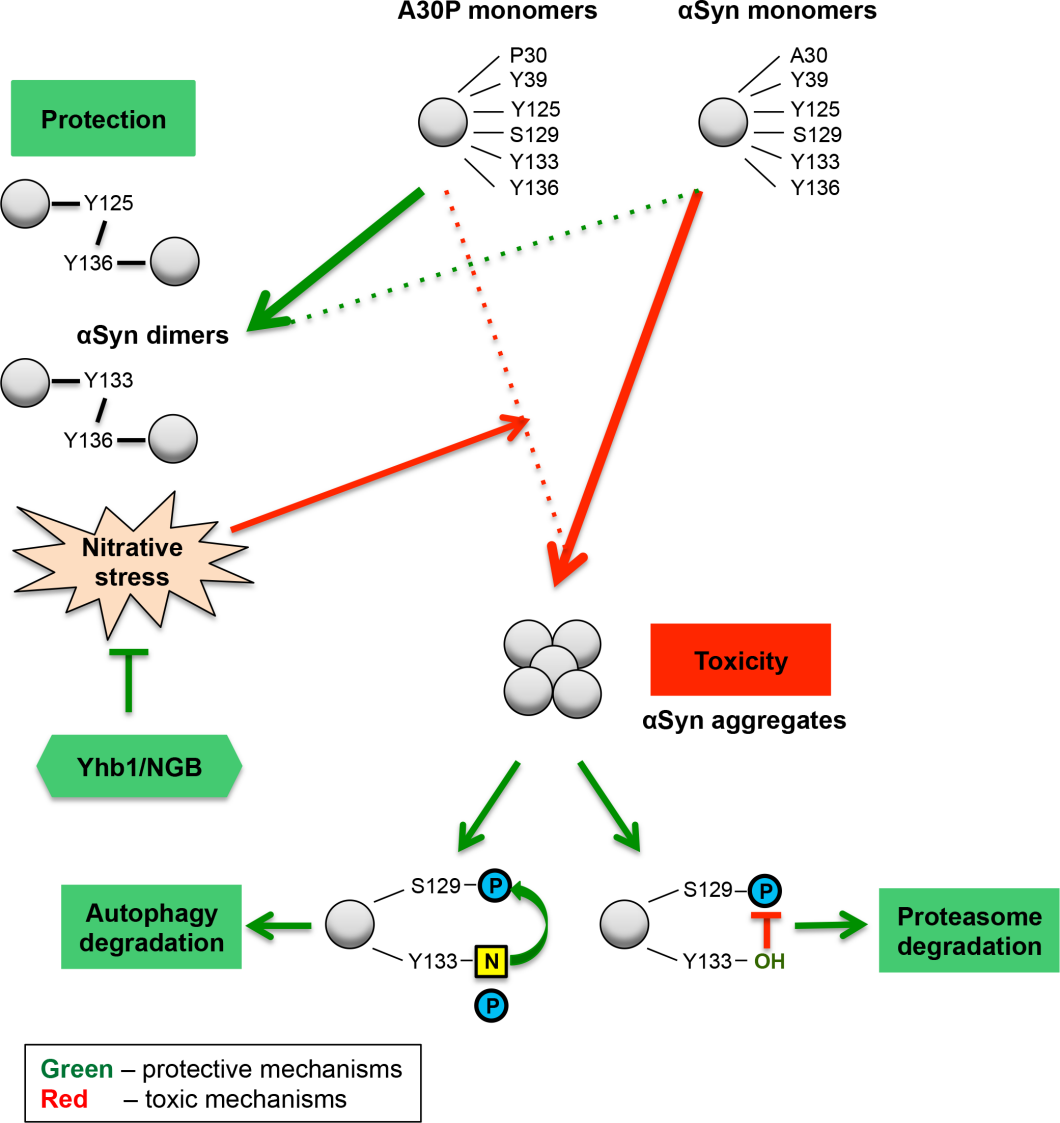
αSyn posttranslational modifications and nitrative stress in yeast. Enhanced intracellular nitrative stress increases the protein nitration level and influences yeast growth and aggregation. The nitration of tyrosine residues acts as trigger for αSyn and A30P toxicity. Wild-type αSyn, which is highly nitrated, inhibits growth and shows a high aggregation rate. A30P is weakly nitrated and therefore, does not inhibit yeast growth and has a low aggregation propensity. Yhb1 and its human homolog NGB protect the cells against accumulation of nitrative species and diminish the aggregate formation. Di-tyrosine crosslinked dimers are formed in reverse correlation to cytotoxicity and do not depend on Yhb1. A30P forms twice as many dimers as the toxic αSyn variant, suggesting that the di-tyrosine crosslinked dimers are not toxic species and are presumably part of a cellular detoxification pathway, sequestering the protein off-pathway of αSyn nucleation. The C-terminal tyrosine modifications have dual effect on the toxicity of the protein. Y133, which is nitrated and phosphorylated, is required for the protective phosphorylation at S129 and for the autophagy degradation of αSyn aggregates. Non-modified Y133 promotes the proteasomal degradation of αSyn aggregates. N: nitration; P: phosphorylation.

Phosphorylation at serine residue S129 represents the major protective phosphorylation site of αSyn which is conserved from man to the baker’s yeast as a eukaryotic Morbus Parkinson cell model. The effect of nitrative modifications of αSyn and their contribution towards αSyn-induced cytotoxicity was investigated. A complex interplay was discovered between modifications of the C-terminal tyrosine residues and S129 phosphorylation (Fig 14). These tyrosine residues of αSyn can be phosphorylated or nitrated with drastic consequences for cellular growth. There is a strong preference of the C-terminus of αSyn for nitration or di-tyrosine formation. Nitration interferes with protective phosphorylation of S129, whereas di-tyrosine formation protects yeast cells. The yeast nitric oxidoreductase Yhb1 as well as its related human protein neuroglobin play protective roles against αSyn aggregation. Yhb1 decreases the nitration level of the A30P variant of αSyn by reducing the accumulation of reactive nitrogen species, resulting that yeast cells can tolerate increased levels of this αSyn variant without significant growth inhibition.

In yeast, expression of αSyn triggers accumulation of chemically reactive molecules such as ROS and RNS that damage the cell by causing oxidative and nitrative stress which in turn contributes to cell death [66]. Nitration reduces the binding affinity of αSyn to lipid vesicles and therefore disrupts αSyn-membrane interaction [87]. Numerous studies show that oxidative injury of αSyn, specifically nitration of tyrosine residues, contributes directly to the pathology of PD. Nitrative stress was proposed to induce αSyn aggregation as well as αSyn-induced pathology [34, 47, 50, 88–90]. However, also opposing influence of nitrated αSyn has been shown [51, 52]. Thus, the effect of nitrative αSyn modifications and their influence on the toxicity and aggregation of αSyn is still controversial.

In this study, we show that PTMs on αSyn tyrosine residues have a dual impact on αSyn mediated growth inhibition of yeast cells. Previous studies suggested that nitration of αSyn might be responsible for the formation of the proteinaceous inclusions observed in PD brains and for the neuronal loss in the *substantia nigra* [34, 88]. Here, we showed that nitration increases the growth defect induced by αSyn in yeast cells. Tyrosine nitration increases aggregation, mitochondrial fragmentation and growth inhibition. We demonstrated a correlation between the growth impairment, mediated by αSyn and A30P and their nitration level. αSyn, which inhibits yeast growth and has a high aggregation level, is strongly nitrated. In contrast, A30P, which is not toxic to yeast and aggregates to a lesser extent, is weakly nitrated.

Notably, we showed that C-terminal tyrosine 133 is required for the protective phosphorylation of αSyn at S129. αSyn C-terminal tyrosines Y125, Y133 and Y136 are in close proximity to S129, which raises the question whether there is an interplay between different PTMs at these residues. S129-phosphorylated αSyn is abundantly found in Lewy bodies [35, 37]. This phosphorylation site is conserved in yeast and can be used by several endogenous kinases [91]. The effects of S129 phosphorylation are complex and the role of this modification on αSyn-induced toxicity and aggregation is still controversial [38]. In yeast, S129 phosphorylation has a protective role against growth impairment and aggregate formation [39–41]. Phosphorylation of the tyrosine residues of αSyn is less explored and the effects of this modification are still unclear, varying from protective to no impact on neurotoxicity and oligomerization [83, 92–97]. We show for the first time that tyrosine 133 is strictly required for phosphorylation at S129 in yeast. Tyrosine 133 can be nitrated or phosphorylated, as demonstrated by immunoblotting and MS analysis. These two PTMs might have opposing roles on the cellular toxicity of the protein. Phosphorylation might prevent tyrosine residues from nitration and *vice versa*. Here, we used tyrosine to phenylalanine exchanges that abolishes both phosphorylation as well as nitration. There are no natural amino acids that mimic the phosphorylation or nitration state of the tyrosine residue, thus restricting the investigation of the contribution of a single posttranslational modification at one and the same tyrosine residue *in vivo*. Our results reveal Y133 as major tyrosine phosphorylation site in yeast, and we observe only insignificant phosphorylation at Y125. Our data reveal a correlation between tyrosine nitration and the cellular S129 phosphorylation level. The wild-type αSyn has significantly higher nitration levels as well as increased protein populations with S129 phosphorylation but similar levels of Y133 phosphorylation when compared to its A30P variant. These results suggest that rather nitration than phosphorylation at Y133 promotes S129 phosphorylation in yeast. Alternatively, nitration at Y133 might change the protein conformation and make S129 more accessible for protein kinases. The protective effect of Y133 was not accompanied with changes in the potential to form aggregates. The discrepancy between the clear protective effect of Y133 on yeast growth without significant effect on inclusion formation suggests that additional yet elusive protective mechanisms exist in the cell, which do not depend on aggregate formation but support cellular survival. This suggests an even more complicated interplay between different α-synuclein modifications. Recently, the role of site-specific nitration of αSyn was investigated using site-specifically nitrated synthetic proteins at Y39 and Y125 [98]. The authors assessed the influence of nitration at Y125 and Y39 on PLK3-mediated *in vitro* phosphorylation at S129 and showed that tyrosine nitration does not prevent recognition of the protein by PLK3 and the subsequent phosphorylation at S129. These results strengthen the link between the C-terminal tyrosine and serine modifications, revealing a complex cross-talk between PTMs with different contributions to the cytotoxicity of αSyn.

Previously, we have shown that autophagy is the major pathway for aggregate clearance in yeast [63]. The phosphorylation state of αSyn influences the clearance mechanism of the protein. Blocking of S129 phosphorylation in yeast leads to impaired aggregate clearance by autophagy [39]. Increased levels of S129 phosphorylation can suppress the defect of impaired αSyn sumoylation by rescuing the autophagic aggregate clearance and promoting the proteasomal clearance of αSyn [41]. Here, we show that posttranslational modifications of the four tyrosine residues, similar to S129 phosphorylation, promote the autophagic clearance of αSyn aggregates. Inhibition of autophagy rendered yeast cells unable to clear αSyn aggregates, however had no effect on the clearance of S129A, 4(Y/F) and Y133F mutants. Interestingly, Y133F mutant could not be degraded upon inhibition of the proteasome. Similarly, proteasome impairment resulted in inefficient clearance of the A30P/Y133F mutant, however the impact was not as strong as in the αSyn Y133F mutant. Therefore, Y133 represents a key determinant for the degradation fate of αSyn. Phospho- and nitro-modifications at Y133 promote the autophagic clearance of the aggregates, whereas the non-modified Y133 protein is directed to the proteasome.

Beside nitration, the reaction between tyrosine and RNS can result in the formation of di-tyrosine bonds, leading to the formation of stable αSyn oligomers, including dimers and higher oligomeric structures. At low peroxynitrite level, di-tyrosine formation outcompetes the reaction of tyrosine nitration [54, 55] indicating a forced reaction to tyrosine nitration under nitrative stress. We observed formation of dimers and oligomers *in vivo* without exposure of yeast cells to nitrating agents; therefore, they represent the consequence of endogenous nitrative stress. Previous studies already demonstrated nitration-induced oligomerization of αSyn [42, 53]. There, treatment with nitrating agent resulted in the formation of αSyn dimers and oligomers crosslinked by di-tyrosine bonds. Using MS, we characterized the tyrosine residues involved in covalent dimer formation and nitration and identified to what extend the different tyrosine residues are involved in the di-tyrosine formation and what are the precise positions of the respective tyrosines. To the best of our knowledge, this is the first characterization of αSyn dimer species, formed *in vivo* and without additional exposure to nitrative agents. Our data reveal strong preference of the C-terminal tyrosine residues for dimer formation with predominant forms including Y136 interacting either with Y125 (Y125-Y136) or with Y133 (Y133-136). Y39 was hardly involved in dimer formation under physiological conditions in yeast or *in vitro* after PON treatment. Recently, *in vitro* studies on αSyn oligomerization and di-tyrosine formation upon treatment with tetranitromethane (TMN) demonstrate a predominant formation of di-tyrosine dimers when Y39 is not available for nitration (Y39F) and suggest that the N-terminal region of αSyn plays a role in TMN-induced di-tyrosine formation of higher order oligomers [98].

There are studies which described an increase of nitration at Y39 of αSyn in an oxidative cellular model of PD [99], whereas we observe resistance of Y39 to 3-NT modification in comparison to the C-terminal tyrosine residues which are nitrated *in vitro* as well as *in vivo*. These results are corroborated by previous findings, where treatment of purified αSyn with PON did not result in nitration of Y39; however, all C-terminal residues were nitrated [100]. Higher nitration levels of αSyn compared to A30P were also detected by mass spectrometry data confirming the results from western blot analyses. There, only one tyrosine residue is nitrated in A30P, whereas three αSyn residues are nitrated.

Our data reveal that αSyn dimers originating from di-tyrosine crosslinking are non-toxic species in contrast to nitrated αSyn. Covalent binding of the di-tyrosines stabilizes the dimeric structures and consequently removes αSyn molecules from aggregation to toxic aggregates. αSyn protein lacking all four tyrosine residues forms less aggregates, however, the aggregate formation is not prevented. This indicates that tyrosine residues are not crucial for *in vivo* assembly of the protein to aggregates and suggests an independent pathogenic mechanism of αSyn aggregation. Similarly, nitration of αSyn was shown to promote formation of stable off-pathway oligomeric species that inhibit αSyn fibrillation [51, 52]. Thus, the formation of stable oligomers under oxidative stress conditions redirects the monomers to oligomers that do not contribute to fibril formation.

Expression of A30P in yeast has different toxicity properties from wild-type αSyn [60, 63]. A30P is located in the cytoplasm, whereas αSyn is delivered to the plasma membrane. Overexpression from a high-copy number plasmid results in formation of A30P fluorescent foci, however, the aggregation is transient and yeast cell growth is not affected. Using the Δ*yhb1* mutant lacking the flavohemoglobin Yhb1, we could demonstrate that nitration plays also a role for A30P and confirmed that nitration increases aggregation and growth inhibition. Deletion of the *yhb1* yeast gene results in a deficient cellular detoxification machinery towards NO and makes A30P as toxic as wild-type αSyn. This shows that the A30P nitration level is a crucial factor for gaining toxicity. Consequently, elimination of its putative NO-detoxifier Yhb1 results in stronger formation of toxic αSyn aggregates. Yhb1 reduced the level of nitrative stress in A30P expressing cells. Moreover, Yhb1 protected the A30P-expressing cells from mitochondrial fragmentation. αSyn-induced fragmentation of mitochondrial structure caused by direct interaction of αSyn with the mitochondrial membranes was already demonstrated [78]. It was proposed that ROS accumulation induced by αSyn expression is an indirect effect due to mitochondrial dysfunction [65]. We found a connection between increased nitrative stress and mitochondrial fragmentation. This suggests a mechanistic model based on the specific ability of αSyn and A30P to form aggregates and damage mitochondria induced by nitrative stress.

Analysis of neuroglobin (*NGB*), the human homologue of *YHB1*, in human cell lines (H4 cells) confirmed that this protein modulates αSyn aggregation. Expression of neuroglobin in mammalian cells reduced the number inclusions per cell. Neuroglobin is expressed primarily in neurons and protects against hypoxic neuronal death and ischemic brain injury [101]. Furthermore, expression of neuroglobin protects against beta-amyloid-induced neurotoxicity in transgenic mice *in vivo* [80]. Recent reports revealed that overexpression of neuroglobin prevents tau hyperphosphorylation at multiple AD-related sites [102]. These data support our findings and imply NGB as a new therapeutic target in PD and other neurodegenerative diseases.

A complex interplay between nitration and phosphorylation of αSyn C-terminal residues, deeply interconnected with nitrative stress, determines the aggregate clearance by autophagy or ubiquitin-dependent 26S proteasome pathways (Fig 14). The proposed model derived from yeast as unicellular eukaryotic cell should provide interesting hints and insights for the study of αSyn posttranslational communications as it happens in the human brain and its connection to oligomer or aggregate formation and clearance.

## Materials and Methods

### Plasmid construction, yeast strains, transformation and growth conditions

Plasmids (Table 2) and *Saccharomyces cerevisiae* strains (Table 3) used in this work are listed below. Human αSyn cDNA sequence and the corresponding A30P sequence were expressed from yeast high expression vector (2μ) under the *GAL1* promoter and *CYC1* terminator as described previously [63]. *YHB1* sequence was amplified on genomic DNA from *Saccharomyces cerevisiae* and *NGB* was amplified on cDNA sequence and cloned into pME2788 low copy vector (*CEN/ARS*) proceeded by *GAL1* promoter and followed by *CYC1* terminator, respectively. The 4(Y/F) αSyn mutant constructs were generated by site-directed mutagenesis using QuickChange II Site-Directed Mutagenesis Kit (Agilent Technologies). Plasmids pME3763, pME3764, pME4095 and pME4101 were used as templates to substitute the four tyrosines (Y39, Y125, Y133, Y136) by phenylalanine. The same plasmids were used as templates to substitute serine 129 by alanine. For growth and microscopy studies, αSyn variants were used that are C-terminally tagged with GFP via the KLID linker [63]. For Ni^2+^-NTA affinity chromatography, αSyn and A30P were C-terminally fused to His_6_-tag using pME3760 and pME3761 as templates. All constructs were verified by DNA sequencing.

**Table 2:** Plasmids used in this study.

Plasmid	Description	Source
pME2788	*pRS413-GAL1-*promoter, *CYC1-*terminator, *HIS3*, *CEN/ARS*, *pUC origin*, *Amp*^*R*^	[104]
pME2795	*pRS426-GAL1-*promoter, *CYC1-*terminator, *URA3*, *2μm*, *pUC origin*, *Amp*^*R*^	[104]
pME3759	pME2795 with *GAL1*::*GFP*	[63]
pME3760	pME2795 with *GAL1*::*SNCA*^WT^	[63]
pME3761	pME2795 with *GAL1*::*SNCA*^A30P^	[63]
pME3763	pME2795 with *GAL1*::*SNCA*^WT^::*GFP*	[63]
pME3764	pME2795 with *GAL1*::*SNCA*^A30P^::*GFP*	[63]
pME4095	pME2795 with *GAL1*::*SNCA*^WT^::6 x *HIS*	[41]
pME4101	pME2795 with *GAL1*::*SNCA*^A30P^::6 x *HIS*	This study
pME4104	pME2788 with *GAL1*::*NGB*	This study
pME4351	pME2788 with *GAL1*::*YHB1*	This study
pME4352	pME2795 with *GAL1*::*SNCA*^WT^Y39/125/133/136F::*GFP*	This study
pME4353	pME2795 with *GAL1*::*SNCA*^WT^Y39/125/133/136F::6 x *HIS*	This study
pME4354	pME2795 with *GAL1*::*SNCA*^A30P^Y39/125/133/136F::6 x *HIS*	This study
pME4355	pME2795 with *GAL1*::*SNCA*^A30P^Y39/125/133/136F::*GFP*	This study
pcDNA3.1		Invitrogen
pME4357	pcDNA3.1 with *CMV*::*NGB*::*mCherry*	This study
pME4088	pME2795 with *GAL1*::*SNCA*^WT^Y125F::*GFP*	This study
pME4451	pME2795 with *GAL1*::*SNCA*^WT^Y39F::*GFP*	This study
pME4452	pME2795 with *GAL1*::*SNCA*^WT^Y39F::6 x *HIS*	This study
pME4453	pME2795 with *GAL1*::*SNCA*^WT^Y125F::6 x *HIS*	This study
pME4454	pME2795 with *GAL1*::*SNCA*^WT^Y125/133/136F::*GFP*	This study
pME4455	pME2795 with *GAL1*::*SNCA*^WT^Y125/133/136F::6 x *HIS*	This study
pME4456	pME2795 with *GAL1*::*SNCA*^WT^S129A::*GFP*	This study
pME4457	pME2795 with *GAL1*::*SNCA*^WT^S129A::6 x *HIS*	This study
pME4460	pME2795 with *GAL1*::*SNCA*^WT^ Y133F::6 x *HIS*	This study
pME4461	pME2795 with *GAL1*:: *SNCA*^WT^ Y133F:: *GFP*	This study
pME4462	pME2795 with *GAL1*::*SNCA*^WT^ Y136F::6 x *HIS*	This study
pME4463	pME2795 with *GAL1*:: *SNCA*^WT^ Y136F:: *GFP*	This study
pME4466	pME2795 with *GAL1*:: *SNCA*^*A30P*^ S129A:: *GFP*	This study
pME4467	pME2795 with *GAL1*:: *SNCA*^*A30P*^ Y133F:: *GFP*	This study

**Table 3:** Yeast strains used in this study.

Strain	Genotype	Source
BY4741	*MATa; his3Δ 1; leu2Δ0; met15Δ0; ura3Δ0*	EUROSCARF
Δ*yhb1*	*BY4741; MATa; his3D1; leu2D0; met15D0; ura3D0; YGR234w*::*kanMX4*	EUROSCARF

*Saccharomyces cerevisiae* strains, BY4741 and Δ*yhb1*, were grown in non-selective medium (YEPD) at 30°C and transformed by standard lithium acetate protocol [103]. For cultivation of the Δ*yhb1* strain, 200 μg/ml G418 were added to the medium. Transformants harboring αSyn constructs were selected on solid Synthetic Complete medium (SC) lacking uracil (SC-Ura) supplemented with 2% glucose for 2 days at 30°C. For growth of cells co-expressing αSyn with *YHB1* and *NGB*, respectively, SC medium lacking uracil and histidine (SC-Ura-His) was used. Expression of αSyn was induced by shifting overnight cultures from 2% raffinose- to 2% galactose-containing medium (*A*_*600*_ = 0.1).

### Spotting assay and growth analysis in liquid culture

To investigate growth on solid medium, cells were pre-grown in selective SC medium containing 2% raffinose. After normalizing the cells to equal densities (*A*_*600*_ = 0.1), 10-fold dilution series were prepared and spotted in a volume of 10 μl on SC-Ura or SC-Ura,-His agar plates supplemented with either 2% glucose or 2% galactose. The growth was documented after incubation for 3 days at 30°C. For growth test in liquid cultures, cell cultures were pre-grown as described above, adjusted to equal densities of *A*_*600*_ = 0.1 and shifted to galactose-containing SC-Ura medium. Optical density measurements of 200 μl cell cultures were performed in 96-well plates for 48 h using a microplate reader (Infinite M200; TECAN Group Ltd). Growth analyses under nitrative stress conditions were performed using either 600 μM or 1 mM DETA-NONOate (Cayman Chemical Company) as NO donor in SC-Ura medium at pH 7.4.

### Fluorescence microscopy, mitochondrial staining and quantifications

Cells were pre-grown in selective SC medium containing raffinose and inoculated in galactose-containing SC medium to an *A*_*600*_ = 0.1. αSyn expression was induced for 6 h and fluorescent images were obtained with 63x magnification using a Zeiss Observer. Z1 microscope (Zeiss) equipped with a CSU-X1 A1 confocal scanner unit (YOKOGAWA), QuantEM:512SC digital camera (Photometrics) and SlideBook 6.0 software package (Intelligent Imaging Innovations). Depending on the fluorescent agent, ssGFP or sdRFP filter were applied. To quantify aggregation, at least 300 cells were counted per strain and experiment and the number of cells displaying αSyn aggregation was referred to the total number of counted cells. To label mitochondria, the cells were incubated for 45 minutes (min) in the presence of 50 nM MitoTracker Red (Molecular Probes, Invitrogen), washed once with fresh medium and imaged. To test yeast for production of ROS, cells pre-grown overnight in raffinose-containing SC medium were transferred to galactose-containing SC medium at *A*_*600*_ = 0.1 and incubated for 6 h at 30°C. After washing the cells, dihydrorhodamine 123 (DHR123) Cayman Chemical Company) was added to a final concentration of 5 μg/ml and the cells were incubated for 1.5 h at 30°C. After washing, the cells were re-suspended in H_2_O and microscopy was performed using RFP filter. To test yeast for RNS production, cells pre-grown overnight in raffinose-containing SC medium were transferred to galactose-containing SC medium at *A*_*600*_ = 0.1. After 6 h induction cells were washed and diluted in PBS buffer, pH 7.5 to *A*_*600*_ = 0.1. DAF-2 DA (Genaxxon bioscience GmbH) was added to a final concentration of 25 μg/ml and cells were incubated for 1 h at 30°C in the dark. Before microscopy, the cells were washed and RNS was visualized using GFP filter.

### Flow cytometry

For testing yeast cells for production of ROS and RNS, flow cytometry was performed. Cells were grown as above. Before measuring, cells were re-suspended in 50 mM trisodium citrate buffer, pH 7.0. Flow cytometry analysis was performed on a BD FACSCANTO II (Becton Dickinson). 100 000 events were counted for each experiment. Data analysis was performed using the BD FACSDIVA software (Becton Dickinson). Representative examples that are shown in the Figures were repeated at least 3 times. Yeast cell membrane integrity was analyzed with PI staining. Yeast cells were incubated with 12.5 μg/ml PI for 30 min. As a positive control, cells were boiled for 10 min at 95°C.

### Promoter shut-off assay and chemical treatments

Yeast cells carrying αSyn were pre-grown in selective SC medium containing 2% raffinose overnight and shifted to 2% galactose-containing selective SC medium to induce the αSyn expression for 4 h. Afterwards, cells were shifted to SC medium supplemented with 2% glucose to shut-off the promoter. 4 h after promoter shut-off, cells were visualized by fluorescence microscopy. The reduction of number of cells displaying αSyn inclusions was recorded and plotted on a graph. To study the lysosome/vacuole degradation pathway (autophagy) phenylmethanesulfonyl fluoride (PMSF) in ethanol (EtOH) was applied to the suspension in a final concentration of 1 mM [105]. For impairment of the proteasomal degradation system Carbobenzoxyl-leucinylleucinyl-leucinal (MG132) dissolved in dimethyl sulfoxide (DMSO) was added to the cell suspension in a final concentration of 75 μM and in parallel, equal volume of DMSO was applied to the cells as a control. For drug treatment with MG132 induction-medium containing galactose and shut-off-medium containing glucose was supplemented with 0.003% SDS and 0.1% proline [106].

### Ni^2+^-NTA affinity chromatography and *In vitro* nitration

Purification of His_6_-tagged recombinant αSyn and A30P was performed using yeast cells carrying the 2μ high-copy vectors pME4095 and pME4101, pME4353, pME4354 and pME2795. Cells cultured overnight in 200 ml selective SC medium supplemented with 2% glucose were collected by centrifugation, washed and inoculated in 1.5 liter selective medium containing 2% galactose for 12 h at 30°C. Cells were lysed by 50 ml 1.85 M NaOH containing 7.5% ß-mercaptoethanol on ice for 10 min. For precipitation, the protein crude extracts were incubated for 30 min in 50 ml 50% trichloroacetic acid (TCA) on ice, washed afterwards with acetone and dissolved in 50 ml buffer A (6 M Guanidine hydrochloride, 100 mM NaH_2_PO_4_, 10 mM Tris-HCl, pH 8.0). The mixture was shaken for 1 h at 25°C, the collected supernatant was calibrated to pH 7.0 with 1 M Tris base (pH 8.5) and supplemented with imidazole to final concentration of 20 mM. Before the protein crude extract was applied to a His GraviTrap column (GE Healthcare Life Science), the columns were washed with 10 ml buffer A containing 20 mM imidazole and equilibrated with 5 ml buffer B (8 M Urea, 100 mM Na_3_PO_4_, 10 mM Tris, pH 6.3). Elution of the protein was carried out using 4 times 1 ml of 200 mM imidazole resolved in buffer B. The protein content of the elution fractions were determined by Bradford protein concentration assay and subjected to western blot analysis. *In vitro* nitration of αSyn was performed using the highly reactive nitrating agent peroxynitrite (PON) (Cayman Chemical Company) in the presence of 0.3 M HCl to adjust the pH value. 20 μl of the protein were mixed with 1 μl of both reagents for approximately 10 seconds.

### Immunoblotting

Yeast cells harboring different αSyn constructs were grown in selective SC medium containing 2% raffinose overnight and transferred to 2% galactose-containing medium for induction of αSyn expression. After expression of αSyn for 6 h, protein crude extracts were prepared and protein concentration was determined via Bradford protein concentration assay. For electrophoretic separations of the protein, 10 μg protein extract was applied to a 12% SDS-polyacrylamide-gel and transferred afterwards onto a nitrocellulose or polyvinylidenflouride (PVDF) membrane. The membrane was incubated with primary antibody diluted in TBST buffer with 5% milk powder overnight. As primary antibodies, rabbit anti α/β/γSyn polyclonal antibody (1:2000, Santa Cruz Biotechnology), mouse anti 3-nitrotyrosine monoclonal (1:1400, Abcam), mouse anti nitro-α/ßSyn (Tyr39) monoclonal antibody (1:2000, MERCK), mouse anti di-tyrosine monoclonal antibody (1:1000, JaICA), mouse anti phospho Ser-129 αSyn antibody (1:2500, Wako Chemicals USA), rabbit anti phospho Tyr-133 αSyn polyclonal antibody (1:1000, Abcam) and mouse anti GAPDH monoclonal antibody (1:5000, Thermo Fisher Scientific) were used. Peroxidase-coupled goat anti mouse (1:2000, Jackson ImmunoResearch Laboratories) or goat anti rabbit (1:5000, Mobitec) immunoglobulins G was applied as secondary antibody.

### Quantifications of Western Blots

Pixel density values for Western Blot quantification were obtained from TIFF files generated from digitized x-ray films (Kodak) and analyzed with the ImageJ software (NIH, Bethesda). Sample density values were normalized to the corresponding loading control. For quantification of the signals, at least three independent experiments were performed. The significance of differences was calculated using Student’s t test or one-way ANOVA test. p value < 0.05 was considered to indicate a significant difference.

### Mass spectrometry analysis

Protein samples were separated by 12% SDS-PAGE. Excised polyacrylamide gel slices of Coomassie stained proteins were digested with the proteases trypsin (Promega) and AspN (Sigma-Aldrich) according to the protocol of Shevchenko [107] and supplier’s instructions. After digestion and peptide elution the samples were resolved in 20 μl 2.8% acetonitrile containing 0.1% formic acid. The single digestions as well as the double digested AspN/tryptic peptides were then analyzed by LC-MS.

Peptides of 1–5 μl sample solution were trapped and washed with 0.07% trifluoroacetic acid 2.6% acetonitrile on an Acclaim PepMap 100 column (100 μm x 2 cm, C18, 3 μm, 100 Å, P/N164535 Thermo Scientific) at a flow rate of 25 μl/min for 5 min. Analytical peptide separation by reverse phase chromatography was performed on an Acclaim PepMap RSLC column (75 μm x 25 cm, C18, 3 μm, 100 Å, P/N164534 Thermo Scientific) running a 40 min gradient from 100% solvent A (0.1% formic acid) to 65% solvent B (80% acetonitrile, 0.1% formic acid) and further to 95% solvent B within 1 min at flow rates of 300 nl/min (Fisher Chemicals). Chromatographically eluting peptides were on-line ionized by nano-electrospray (nESI) using the Nanospray Flex Ion Source (Thermo Scientific) at 2.4 kV and continuously transferred into the mass spectrometer. Full scans within m/z of 300–1850 were recorded with the Orbitrap-FT analyzer at a resolution of 30.000 with parallel data-dependent top 10 MS2-fragmentation in the LTQ Veleo Pro linear ion trap. LC-MS method programming and data acquisition was performed with the software Xcalibur 2.2 (Thermo Fisher). MS/MS2 data processing for protein analysis and PTM identification was done with the Proteome Discoverer 1.4 (PD, Thermo Scientific) software using the SequestHT search engine (Thermo Scientific) and *Saccharomyces cerevisiae* protein database extended by the most common contaminants with the following criteria: peptide mass tolerance 10 ppm, MS/MS ion mass tolerance 0.8 Da, and up to two missed cleavages allowed. Only high confident peptides with a false discovery rate less than 0.01 were considered.

### Identification of crosslinked peptides

The MS data of crosslinked peptides were analyzed with StavroX2.3.4.5 [73]. MS data in the Mascot generic file (mgf) format containing all MS/MS data of precursor ions were loaded into the program. The following parameters were used for the StavroX analysis: (i) cleavage sites: C-terminal: K, R; N-terminal: D; (ii) number of missed cleavages = 2; (iii) variable modifications: oxidation of methionine; nitration of tyrosine; cysteine-to-cysteine acetamid; (iv) mass of crosslinker: -H2; (v) crosslinks only between two tyrosines; (vi) precision precursor comparison = 10 ppm.

The false-positive rate was evaluated by decoy analysis using the reversed protein sequence. The frequency of occurrence of candidates from the data analysis and decoy analysis was compared for each sample. Only scores with decoy frequencies below 8% of the data frequency were considered as possible crosslinks. The data were filtered for unique scans and each scan was considered only once with its highest score. Since multiple tyrosine residues are located on one and the same peptide, different combinations of crosslinked peptides with equal masses were possible. For each scan the crosslinked tyrosine dimers were assigned according to the score calculated by the program based on the fragment ions series.

### Cell culture

Neuroglioma (H4) cells were plated 24 h prior to transfection, in 12-well plates (Costar). Cells were transfected with FuGENE6 Transfection Reagent (Promega) using equal amounts of plasmid DNA encoding for αSyn, synphilin-1 [108] and Neuroglobin-mCherry, or the empty mammalian expression vector pcDNA 3.1, according to the manufacturer’s instructions.

### Immunocytochemistry

48 h after transfection, cells were washed with PBS and fixed with 4% paraformaldehyde for 10 min at room temperature (RT). After washing with PBS, cells were permeabilized with 0.5% Triton X-100/PBS (Sigma-Aldrich) for 20 min at RT, and blocked in 1.5% normal goat serum (PAA)/PBS for 1 h. Cells were incubated with a mouse anti αSyn antibody (1:1000, BD Transduction Laboratory) overnight and then with a secondary antibody (Alexa Fluor 488 donkey anti-mouse IgG) for 2 h at RT. Finally, cells were stained with Hoechst 33258 (1:5000 in PBS, Life Technologies- Invitrogen) for 5 min and maintained in PBS prior to epifluorescence microscopy.

### Quantification of αSyn inclusions and cytotoxicity test in H4 cells

Transfected cells were scored based on the αSyn inclusions pattern and classified into: cells without inclusions, less than ten inclusions (<10 inclusions), and more than ten inclusions (≥10 inclusions), as described [61]. The total number of transfected cells was expressed in percentage, as the average from three independent experiments. The lactate dehydrogenase (LDH) cytotoxicity assay (Roche Diagnostics) was performed according to the manufacturer’s instructions. Growth media from cells were applied in triplicates in a 96-well plate, in a ratio 1:1 with the reaction mixture. The measurements were performed in a TECAN Infinite 200 Pro plate reader at 490 nm. The percentage of toxicity was calculated as indicated by the manufacturer.

### Statistical analysis

Data were analyzed using GraphPad Prism 5 (San Diego, California, USA) Software and were presented as mean ± SEM of at least three independent experiments. The significance of differences was calculated using Students t-test, one-way ANOVA test with Bonferroni’s multiple comparison test or Dunnett’s multiple comparison test. P value < 0.05 was considered to indicate a significant difference.

## Supporting Information

S1 Fig MS2 analysis of cross-linked peptides. (A) Exemplary fragment ion MS2 spectrum of the crosslink between Y133 and Y136 for αSyn dimers. y-ions of the crosslinked peptides are represented in blue, while b-ions are represented in red. Fragmentation sites are indicated in the amino acid sequence. (B) Exemplary fragment ion MS2 spectrum of the crosslink between Y125 and Y136 of A30P dimers.
(PDF)

S2 Fig Forward scatter (FSC) and Propidium Iodide (PI) fluorescence intensity of cells assessed with flow cytometry analysis. Cells expressing different αSyn variants and GFP (control) after 20 h induction of expression were stained with 12.5 μg/ml PI for 30 min. Shown is one representative result from at least four independent experiments.
(PDF)
