# Supplementary material for: C-Terminal Tyrosine Residue Modifications Modulate the Protective Phosphorylation of Serine 129 of α-Synuclein in a Yeast Model of Parkinson's Disease
Source: PLoS Genet. 2016 Jun 24;12(6):e1006098. doi: 10.1371/journal.pgen.1006098 (PMC4920419; doi:10.1371/journal.pgen.1006098)
Supplement: S1 Fig — (A) Exemplary fragment ion MS2 spectrum of the crosslink between Y133 and Y136 for αSyn dimers. y-ions of the crosslinked peptides are represented in blue, while b-ions are represented in red. Fragmentation sites are indicated in the amino acid sequence. (B) Exemplary fragment ion MS2 spectrum of the crosslink between Y125 and Y136 of A30P dimers. (PDF) [file pgen.1006098.s001.pdf]

A

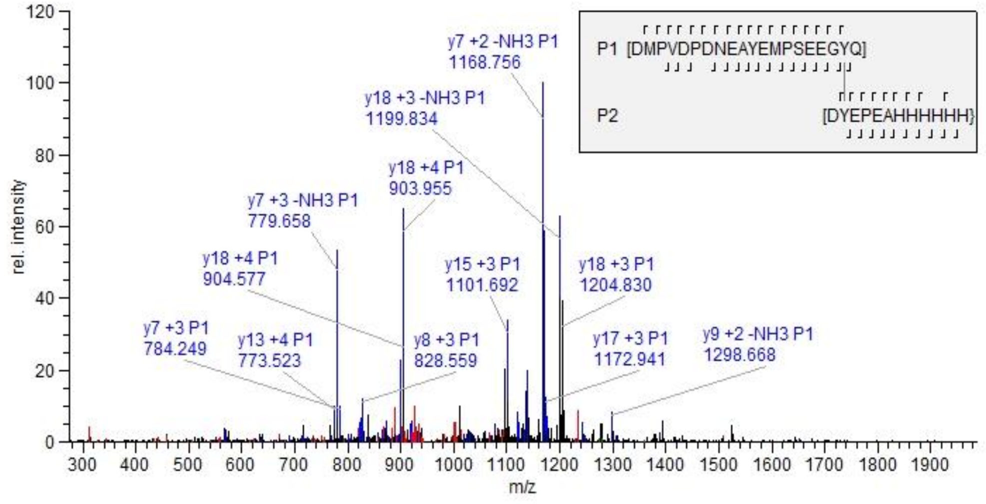

B

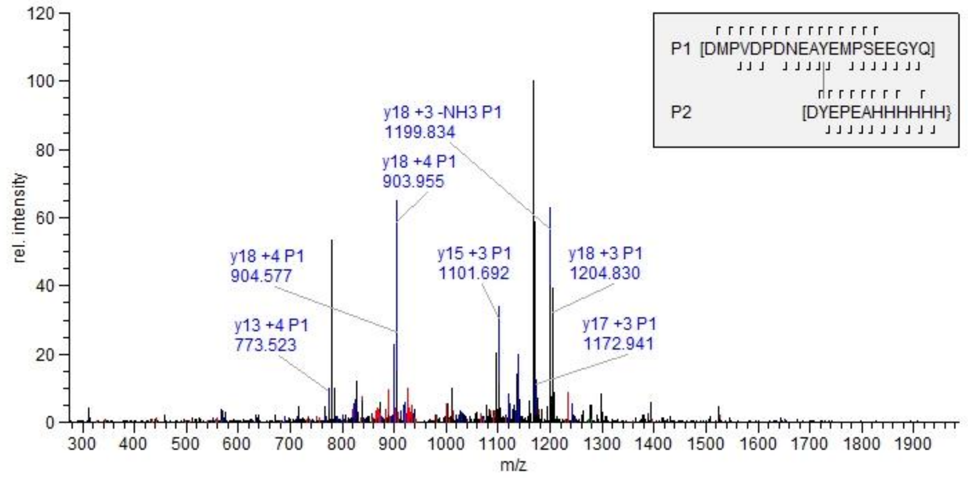

S1 Fig. MS2 analysis of cross-linked peptides. (A) Exemplary fragment ion MS2 spectrum of the crosslink between Y133 and Y136 for αSyn dimers. y-ions of the crosslinked peptides are represented in blue, while b-ions are represented in red. Fragmentation sites are indicated in the amino acid sequence. (B) Exemplary fragment ion MS2 spectrum of the crosslink between Y125 and Y136 of A30P dimers.
