# Supplementary material for: C-Terminal Tyrosine Residue Modifications Modulate the Protective Phosphorylation of Serine 129 of α-Synuclein in a Yeast Model of Parkinson's Disease
Source: PLoS Genet. 2016 Jun 24;12(6):e1006098. doi: 10.1371/journal.pgen.1006098 (PMC4920419; doi:10.1371/journal.pgen.1006098)
Supplement: S2 Fig — Cells expressing different αSyn variants and GFP (control) after 20 h induction of expression were stained with 12.5 μg/ml PI for 30 min. Shown is one representative result from at least four independent experiments. (PDF) [file pgen.1006098.s002.pdf]

PI – 20 h

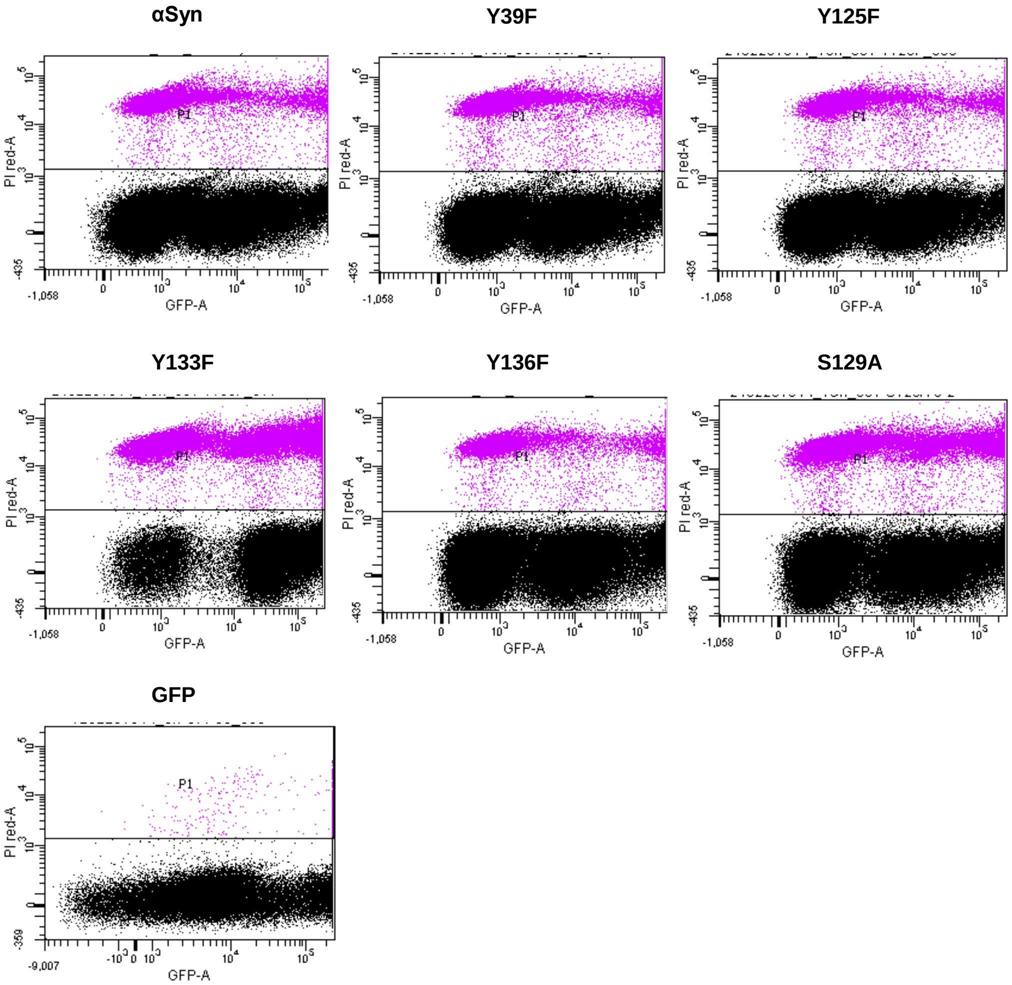

S2 Fig. Forward scatter (FSC) and Propidium Iodide (PI) fluorescence intensity of cells assessed with flow cytometry analysis. Cells expressing different αSyn variants and GFP (control) after 20 h induction of expression were stained with 12.5 μg/ml PI for 30 min. Shown is one representative result from at least four independent experiments.
